# Differential DNA methylation and transcription profiles in date palm roots exposed to salinity

**DOI:** 10.1371/journal.pone.0191492

**Published:** 2018-01-19

**Authors:** Ibtisam Al-Harrasi, Rashid Al-Yahyai, Mahmoud W. Yaish

**Affiliations:** 1 Department of Biology, College of Science, Sultan Qaboos University, Muscat, Oman; 2 Department of Crop Sciences, College of Agricultural and Marine Sciences, Sultan Qaboos University, Muscat, Oman; Purdue University, UNITED STATES

## Abstract

As a salt-adaptive plant, the date palm (*Phoenix dactylifera* L.) requires a suitable mechanism to adapt to the stress of saline soils. There is growing evidence that DNA methylation plays an important role in regulating gene expression in response to abiotic stresses, including salinity. Thus, the present study sought to examine the differential methylation status that occurs in the date palm genome when plants are exposed to salinity, and to identify salinity responsive genes that are regulated by DNA methylation. To achieve these, whole-genome bisulfite sequencing (WGBS) was employed and mRNA was sequenced from salinity-treated and untreated roots. The WGBS analysis included 324,987,795 and 317,056,091 total reads of the control and the salinity-treated samples, respectively. The analysis covered about 81% of the total genomic DNA with about 40% of mapping efficiency of the sequenced reads and an average read depth of 17-fold coverage per DNA strand, and with a bisulfite conversion rate of around 99%. The level of methylation within the differentially methylated regions (DMRs) was significantly (*p* < 0.05, *FDR* ≤ 0.05) increased in response to salinity specifically at the mCHG and mCHH sequence contexts. Consistently, the mass spectrometry and the enzyme-linked immunosorbent assay (ELISA) showed that there was a significant (*p* < 0.05) increase in the global DNA methylation in response to salinity. mRNA sequencing revealed the presence of 6,405 differentially regulated genes with a significant value (*p* < 0.001, *FDR* ≤ 0.05) in response to salinity. Integration of high-resolution methylome and transcriptome analyses revealed a negative correlation between mCG methylation located within the promoters and the gene expression, while a positive correlation was noticed between mCHG/mCHH methylation rations and gene expression specifically when plants grew under control conditions. Therefore, the methylome and transcriptome relationships vary based on the methylated sequence context, the methylated region within the gene, the protein-coding ability of the gene, and the salinity treatment. These results provide insights into interplay among DNA methylation and gene expression, and highlight the effect of salinity on the nature of this relationship, which may involve other genetic and epigenetic players under salt stress conditions. The results obtained from this project provide the first draft map of the differential methylome and transcriptome of date palm when exposed to an abiotic stress.

## Introduction

Soil salinity is one of the most serious environmental obstacles affecting the growth and the productivity of different plant species [1]. By the year 2050, it is expected that more than 50% of arable land will become unsuitable for agricultural uses due to salinization [2]. Therefore, production of salt-tolerant plants is an urgent need necessary to minimize this problem. However, an understanding of the mechanisms underlying salinity adaption in plants is a crucial step toward approaching this target [3].

Plants have evolved complex mechanisms to cope with salinity stress as a multigenic trait, including selective ion uptake and exclusion, synthesis of compatible solutes, photosynthesis and energy metabolism control, toxic ion compartmentalization, anti-oxidative enzyme accumulation, cell structure modification and hormonal regulation [4, 5]. Salinity adaptation involves various physiological processes; however, the efficiency of these processes in salinity tolerance is not only based on the presence of the required genes, but also on the presence of an appropriate pattern of gene expression, a procedure which is controlled in the cell by various epigenetic factors [6].

Epigenetic changes involve three main mechanisms: DNA methylation, histone modifications, and RNA-mediated gene silencing [7]. Cytosine methylation results from the addition of a methyl group (CH_3_) to the carbon 5-position of cytosine bases (5mdC) [8–10]. This process plays a crucial role in genomic imprinting, as well as in gene expression regulation, transposon silencing and transgenerational epigenetic memory [11–14]. In mammalians and plants, DNA methylation occurs in mCG dinucleotide sequence context, as well as in mCHG and mCHH sequence contexts, where H stands for all nucleotides except guanine [15–17]. The cytosine methylation within symmetrical mCG is maintained by the MET1 methyltransferase (DNMT1) [18] and methylation of symmetrical mCHG is maintained by CHROMOMETHYLASE 3 (CMT3) methyltransferase, while asymmetrical mCHH methylation is maintained by persistent *de novo* methylation [19].

There is an important correlation between DNA methylation status and gene expression when plants are exposed to salinity [20–23]. However, the impact of DNA methylation on gene expression varies based on the location of methylated cytosines within the gene body and the methylated sequence contexts [24]. For example, while mCG methylation within the promoter inhibits gene expression [25], the same methylation pattern within the exons enhances gene expression in some species [8, 18, 25, 26].

The date palm (*Phoenix dactylifera* L.) is a relatively salt-tolerant plant. However, few reports have studied the genes and the mechanisms that code salinity tolerance [27–30]. Despite their potential importance in gene regulation under salinity, epigenetic factors including DNA methylation have not yet been investigated in date palms.

DNA methylation changes in response to salinity have previously been studied in different plant species using various molecular techniques, including the methyl-sensitive amplification polymorphism (MSAP) [31–33]. The outcomes of these studies provided evidence about the importance of this epigenetic process in controlling a plant’s response to salinity. However, the detection of differential methylation patterns in response to salinity based on the construction of a single-base resolution methylome has not yet been reported in date palms.

In this study, we hypothesized that cytosine methylation is differentially occurring in the genome when a date palm is prolonged exposed and then adapted to salinity stress. These changes can distinctively affect the expression level of some genes, including those that are associated with salinity tolerance mechanisms. Therefore, the main aims of this study were to explore the patterns of the DNA methylation across the genome of plants grown under control conditions, and to investigate the alterations in the DNA methylation that would ensure the response to salinity, based on a single nucleotide resolution. This study also was aimed to correlate the cytosine methylation with the transcriptome abundance at global levels. This includes those transcripts that have potential association with salinity tolerance mechanisms.

The results showed that the date palm methylome is heavily modulated by salinity treatment and that these modulations were statistically correlated with gene expression patterns.

## Materials and methods

### Plant materials, growth conditions and photosynthetic measurements

Date palm (*Khalas*, a moderately salinity-tolerant cultivar) seeds, derived from a single source of pollen grains, were thoroughly washed with tap water then surface-sterilized in 75% ethanol for 5 minutes. The ethanol traces were washed out by rinsing the seeds four times with sterile distilled water. Thereafter, the seeds were soaked overnight in sterile water at 37°C. The next day, the seeds were mixed with sterilized moist vermiculite and incubated at 37°C in the dark for one week. The germinated seeds were transferred to 2L pots containing peat moss and sand mixture (1:1, *v/v*). Seedlings were grown under controlled conditions of a 16/8-hour light/dark cycle, 350μE m^−2^ s^−1^ light intensity, 35/30°C day/night temperature and 60% humidity. The control treatment group was irrigated to field capacity with distilled water twice a week. The salt stress was gradually applied at an increment of 50 mM every 10 days, to a final concentration of 300 mM NaCl. The 300 mM NaCl treatment lasted for 20 days. The electrical conductivity (EC) of the soil was used as an indication of salinity. The level of soil salinity was measured three days after each salt application using the Em50 Digital Data Logger (Decagon Devices, WA, USA). FluorPen FP100 (Photon System Instruments, Czech Republic) was used to measure chlorophyll fluorescence-related parameters. Net photosynthetic rate (*A*), internal CO_2_ concentration (*ci*), stomatal conductance (*gs*) and transpiration (*E*) were measured using the LCpro-SD Advanced Photosynthesis Measurement System (ADC Bioscientific Ltd., UK).

### Root analysis

Roots of the NaCl-treated and untreated seedlings were scanned, and some phenotypic parameters such as root length, diameter, total surface area and the number of root tips were analyzed using the WinRHIZO (RH-R XLR STD) software (version 5.0, Regent Instruments, Inc., Quebec, QC, Canada).

### DNA and RNA extraction

Root samples of eight seedlings grown either under control or salinity treatments were harvested, pooled and used for total DNA and RNA extraction. Root tissues were crushed in a pre-chilled mortar to fine powder in liquid nitrogen and the genomic DNA was extracted from the grinded samples as previously described [34]. Total RNA was extracted from the same plant tissues, following a protocol described by Xiao et al. [35].

### Whole genome bisulfite sequencing (WGBS)

DNA and RNA samples were analyzed using Zymo Research CORP next generation sequencing facilities (Irvine, CA, USA), following the previously described strategy [36]. Genomic DNA (500 ng) was fragmented with dsDNA Shearase Plus enzyme (2 units). The resulted fragments were further processed in order to construct Methyl-MaxiSeq libraries. The DNA fragments were blunted to sticky ends using the End-It DNA End-Repair Kit. Subsequently, terminal A(s) tails were added to the 3’ end of the sticky-ended fragments while methylated adaptors, which contain 5’-methylcytosine instead of cytosine, were ligated into the 5’ end. The constructed libraries were underwent a bisulfite treatment, using the EZ DNA Methylation Kit (Zymo Research, Irvine, CA, USA), and the DNA fragments were amplified using specific amplification conditions as previously described [37]. Finally, the bisulfite-treated libraries were sequenced using the Illumina Hiseq platform to generate 50 bp paired-end reads on the Illumina HiSeq 1500.

### Processing and alignment of the bisulfite-sequenced libraries

Bisulfite-sequenced reads were identified using standard Illumina base-calling software. After removal of the duplicate reads, unique best alignments were retained using Bismark version 0.14.3 software [38] and the genome coverage was calculate using Bedtools software version 2.26.0 [39]. A non-directional parameter was applied while running the alignment. In order to calculate the conversion efficiency of cytosines after treatment of DNA with sodium bisulfite, filtered reads were first aligned to the plastid genome of *Phoenix dactylifera* L. (Genbank accession number GU811709) [40]. Subsequently, the reads were aligned to the available reference date palm genome in the database (Genbank accession number PRJNA249070) [41].

### Identification of differentially methylated sites (DMSs)

The methylation ratio of each site was calculated by dividing number of methylated cytosine(s) by the total number of cytosine(s) found in the reads and covered at the specific site. Only sites covered by at least five sequence reads were considered for further analysis. The differential methylation ratio due to salinity treatment was measured by subtracting the methylation ratio obtained from the control sample, from the methylation ratio obtained from the salt-treated sample at each 5mdC site. Significant differences in methylation ratios between treated and control samples were statistically determined by Fisher’s exact test (*p* < 0.05, *FDR* ≤ 0.05). The *p* value was then adjusted to *q* value using Benjamini-Hochberg correction for multiple testing. Accordingly, differentially methylated sites (DMSs) of the three sequence contexts (mCG, mCHG and mCHH) were classified based on the value of the difference in the methylation ratios into the following categories: DMSs were classified as “strongly hypermethylated” if the ratio difference was between +33% and 100%, and “hypermethylated” if the ratio difference was less than +33% but above 0%; however, they were considered “strongly hypomethylated” if the ratio difference was between ‒33% and ‒100%, and “hypomethylated” if the ratio difference was less than ‒33% but below 0%.

### Identification of differentially methylated regions (DMRs)

The chromosomes that passed the *p* -value < 0.05 filter, from the methylation site files for each methylation context was selected for further analysis. Subsequently, 50-bp window sliding was used to obtain the DMRs in only those chromosomes that passed filter. For the methylation ratio for a region, the methylation ratio of each site (5X minimum read coverage) within the region of each sample was first calculated, then the values was averaged for the region and subsequently the difference of the average methylation ratio between the control and salinity-treated samples was considered as the methylation difference for the region. For *p* -value calculation, a fisher test was used. Unmethylated count was obtained from the methylation ratio and total count for a region for both samples. Based on this information, a 2 by 2 table was built to calculate a two-tailed *p* -value for each region.

The overall change (increase) in methylation percentage upon exposure to salinity was calculated based on the following formula: ((Hypermethylated DMRs ‒ Hypomethylated DMRs)/ Total Significant DMRs))*100.

### RNA Sequencing and transcriptome analysis

After rRNA depletion, Illumina HiSeq 50 bp single-end reads from the two-pooled RNA samples were constructed and the resulted data were processed following the manufacturer’s instructions (Illumina). The re-sequencing strategy was used to determine the expression level where the short reads were aligned to the *P*. *dactylifera* L. reference genome (genbank accession number PRJNA249070) using TopHat software (version 2.0.13). Cufflinks software (version 2.2.1) was used for transcript assembly and differential expression, and cummeRbund software (version 2.0.0) was used for visualization of differential analysis. The expression level was normalized by considering the fragments per kilobase of exon model per million mapped fragments (FPKM) value. Differentially expressed genes (DEGs) with a significant value (≥ 2-fold changes, *p*-value ≤ 0.001, *FDR ≤* 0.05) were selected for further analysis. To account for the computational necessity of applying a logarithmic transformation to the expression data, any no expression value (0.0) was replaced with a pseudocount (0.158882), which was calculated based on a tenth of the value of the lowest value among the expression data of the two samples. Subsequently, differential gene expression value was obtained using the formula: log_2_ (FPKM of treated/FPKM of control).

### Identification of differentially methylated genes (DMGs)

In this analysis, the region comparison was carried out against the known genes in the annotation file, resulted from the RNA sequencing. The methylation ratios across the entire gene for each sample were calculated based on the methylation ratios of sites that are covered within the coordinates for each gene and have a minimum coverage of 5X. For each sample, the average of the methylation ratios for each site was assigned as the average methylation ratio of the gene for that sample. The methylation differences for the gene are the average of this methylation ratio. Since the total count for the entire gene for each sample and the average methylation ratio were obtained, a fisher test was run and a *p*-value for each gene was generated. The fisher calculation is similar to the one for the DMRs.

To investigate the correlation between the expression level and the methylation status, the expression data were first grouped based on the mapping location within the different gene features (promoter, exon, and intron regions). The DMGs with no methylation value were replaced by a pseudocount (2.75 X 10^‒7^), which was calculated as the tenth of the lowest value among the methylation ratios obtained from the samples. Pearson’s correlation was calculated between the log_2_ of mRNA expression (FPKM) values and the log_2_ of the methylation values using IBM SPSS Statistics 21. The correlation test applied for both the control and the treated samples.

### Functional Annotation and analysis of DMGs

The DMGs containing mCG, mCHG or mCHH sequence contexts were functionally annotated using the Blast2GO PRO software package [42]. Only DMGs of differential gene expression values were included in this analysis. The annotation of the transcript was based on similarity with protein-coding mRNA sequences available in the protein databases. The protein sequences were classified into the categories of biological processes (BP), cellular components (CC) and molecular functions (MF) using gene ontology (GO) annotation. Differential functional enrichment analysis between the upregulated test group and the downregulated reference group were identified using Fisher’s exact test, based on *p* ≤ 0.05. The coded enzymes were mapped to the metabolic pathways using the Kyoto Encyclopedia of Genes and Genomes (KEGG) tools [43], which are implemented within the Blast2GO PRO software.

### Quantification of global DNA methylation (5mdC) using mass spectrometer

Global genomic quantification of 5-hydroxymethyl-2′-deoxycytidine (5hmdC) and 5mdC was carried out using the mass spectrometer available at the Zymo Research, Irvine, CA, USA. DNA samples (400 ng) were digested to single nucleoside using DNA Degradase Plus (Zymo Research, USA). 5hmdC and 5mdC were then quantified by an SRM-based mass spectrometry assay. The assay quantified the concentration of 5mdC and 5hmdC as a percentage of 2′-deoxyguanosine (dG), the internal standard [44]. The calibration ranges (using 40 pmol of dG) were between 0 and 2.5% and between 0 and 25% for 5hmdC and 5mdC, respectively. Each DNA sample was run in triplicate.

### Determination the global 5mdC level using enzyme-linked immunosorbent assay (ELISA) based colorimetric assay

The global (5mdC) level was quantified using DNA samples extracted from roots grown under control and salt stress conditions. Four biological replicates per treatment were used in this analysis. The quantification was carried out using the MethylFlash Methylated DNA 5mdC Quantification Kit (Epigentek Group Inc., Farmingdale, NY, USA), according to the manufacturer’s instructions.

The sequence DNA and RNA read data generated in this report were deposited in the read data archive of the NCBI under the sequence read project (SRP) numbers SRP108055 and SRP108259, respectively.

## Results

### Effect of salinity on the growth, photosynthetic gas exchange and root development

**Fig 1:**
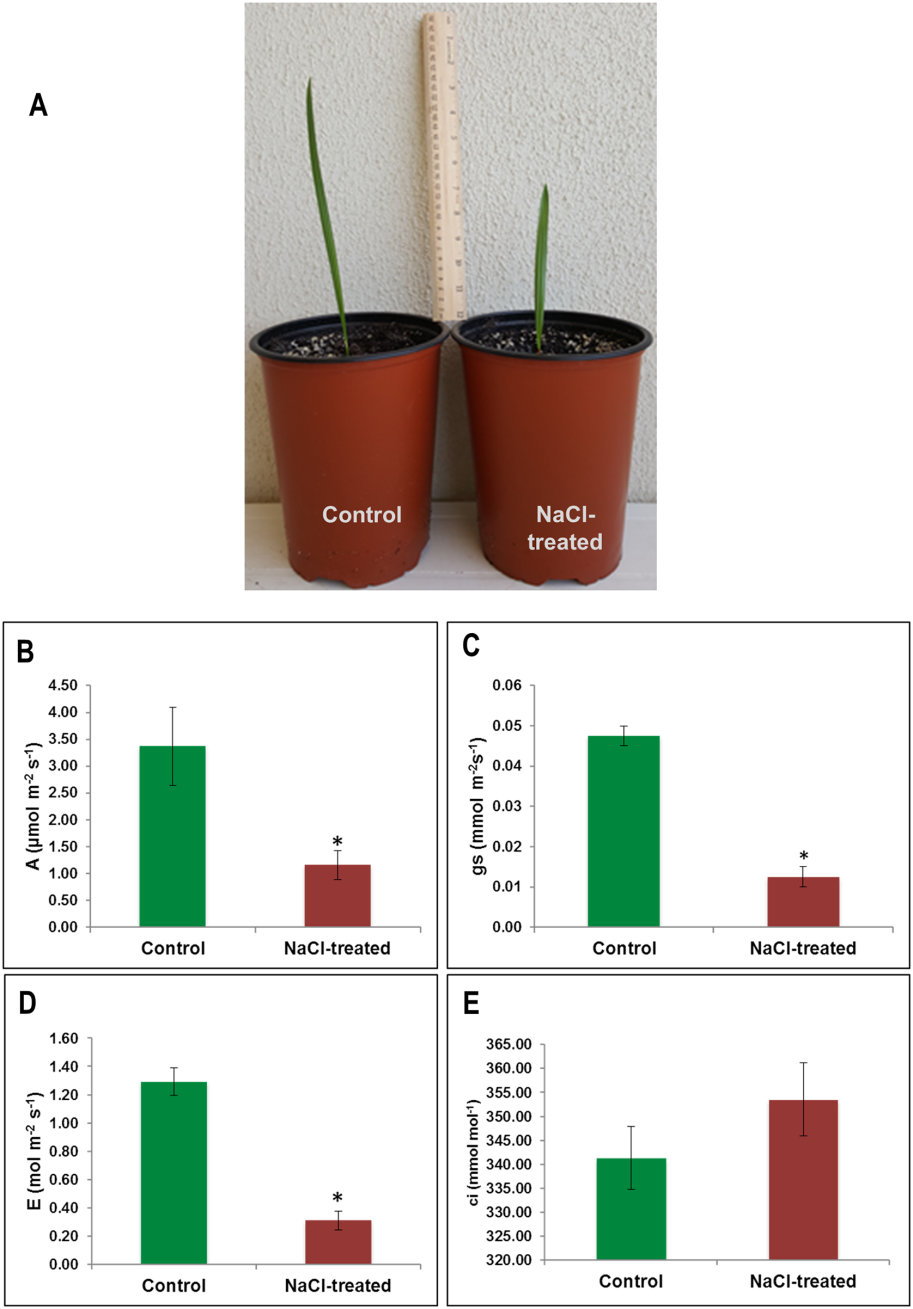
The effect of salinity treatment on photosynthesis performance (A), photosynthesis rate (*A*) (B), stomatal conductance (*gs*) (C), transpiration rate (*E*) (D), and internal CO_2_ concentration (*ci*). The values are means (n = 4) and error bars represent SE (*p* < 0.05). Significant differences are marked by an asterisk.

Photosynthesis performance was used in this project as an indication of plant health status upon salinity treatment. At the end of the salinity treatment, the average electrical conductivity of the soil of the control and NaCl treatment pots was 1.12 ± 0.06 (mean ± SD) and 18.03 ± 0.351 (mean ± SD) dSm^-1^, respectively. A morphological difference between two treatment-groups’ seedlings was noticed with respect to leaf length (Fig 1A). Salt treatment caused significant (*p* < 0.05) reduction of the photosynthetic rate (*A*) (Fig 1B), stomatal conductance (*gs*) (Fig 1C) and transpiration rate (*E*) (Fig 1D) in date palm seedlings. However, there was an insignificant increase in the internal carbon dioxide concentration (*ci*) under salt stress (Fig 1E).

**Fig 2:**
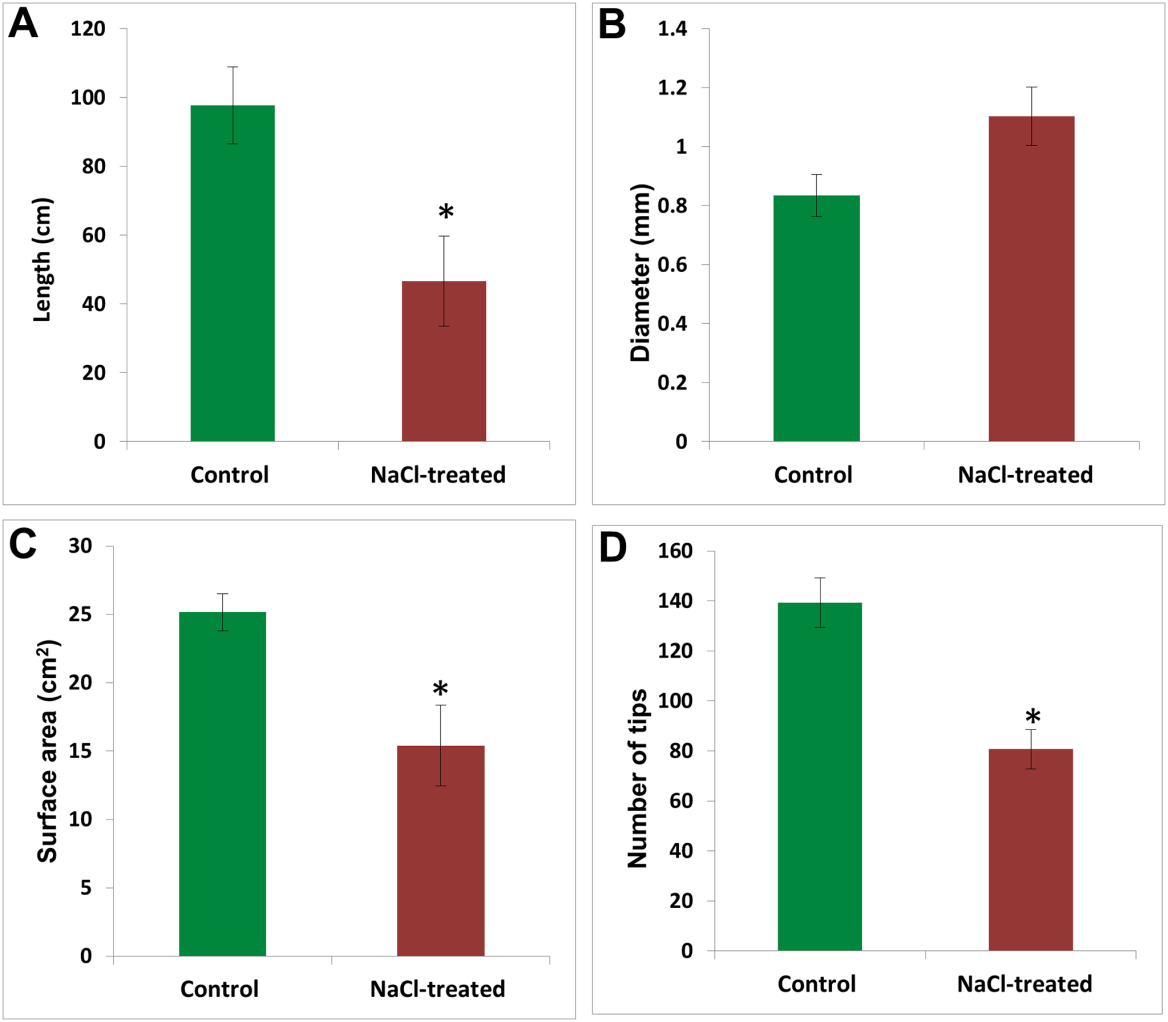
The effect of salinity on root growth, which is measured as root length (A), diameter (B), surface area (C), and number of tips (D). The values are means (n = 4) and error bars represent SE (*p* < 0.05). Significant differences are marked by an asterisk.

Salinity showed a negative effect on the root system (Fig 2). This effect was demonstrated by a significant (*p* < 0.05) reduction in the root length (Fig 2A), surface area and number of root tips (Fig 2C and 2D). The salinity however, had a positive impact on the root diameter (Fig 2B).

### WGBS revealed alterations in the methylation levels upon exposure to salinity stress

After bisulfite conversion, the whole genomes were sequenced using next generation sequencing tools. This type of DNA sequencing has the ability to detect the sites of the methylated cytosine as well as their relative abundance. The bisulfite conversion rates of the genome isolated from untreated (control) and treated plants were 99% and 98%, respectively. The plastid genome of date palm was used to determine the conversion efficiency because it is widely accepted that plastid genomes included methylation free cytosines [45], and this approach is commonly used in plant methylome studies [46].

The WGBS yielded 324,987,795 and 317,056,091 reads from the sequencing of the genomes isolated from the control and the salinity-treated plants, respectively (Table 1). Among these, 19,209,086 and 19,185,920 were aligned to unique positions of mCGs on the genomes extracted from the control and salinity-treated plants, respectively. The WGBS covered around 40% of the total genomic cytosines with an average read depth of 17-fold coverage per each DNA strand (Table 1). The percentage of genome coverage was 80.6% and 80.8% for the control and the salinity-treated samples, respectively. The analysis also revealed the presence of 25,155,548 mCHG and 120,635,111 mCHH sites in the genome isolated from the control plants. However, the same analysis showed the presence of 25,134,635 mCHG and 120,591,563 mCHH sites in the genome isolated from salinity-treated plants (Table 1).

**Table 1:** WGBS analysis of DNA samples extracted from roots grown under control (0 mM NaCl) and the saline (300 mM NaCl) conditions.

Sample	Total Read pairs	Mapping efficiency	mCGs	mCHG	mCHH	Average coverage	Conversion rate
**Control**	324,987,795	40%	19,209,086	25,155,548	120,635,111	17X	99%
**NaCl-Treated**	317,056,091	41%	19,185,920	25,134,635	120,591,563	17X	98%
**Total**	**642,043,886**		**38,395,006**	**50,290,183**	**241,226,674**		

### WGBS revealed an increase in the significantly methylated 5mdC in response to salinity treatment

**Fig 3:**
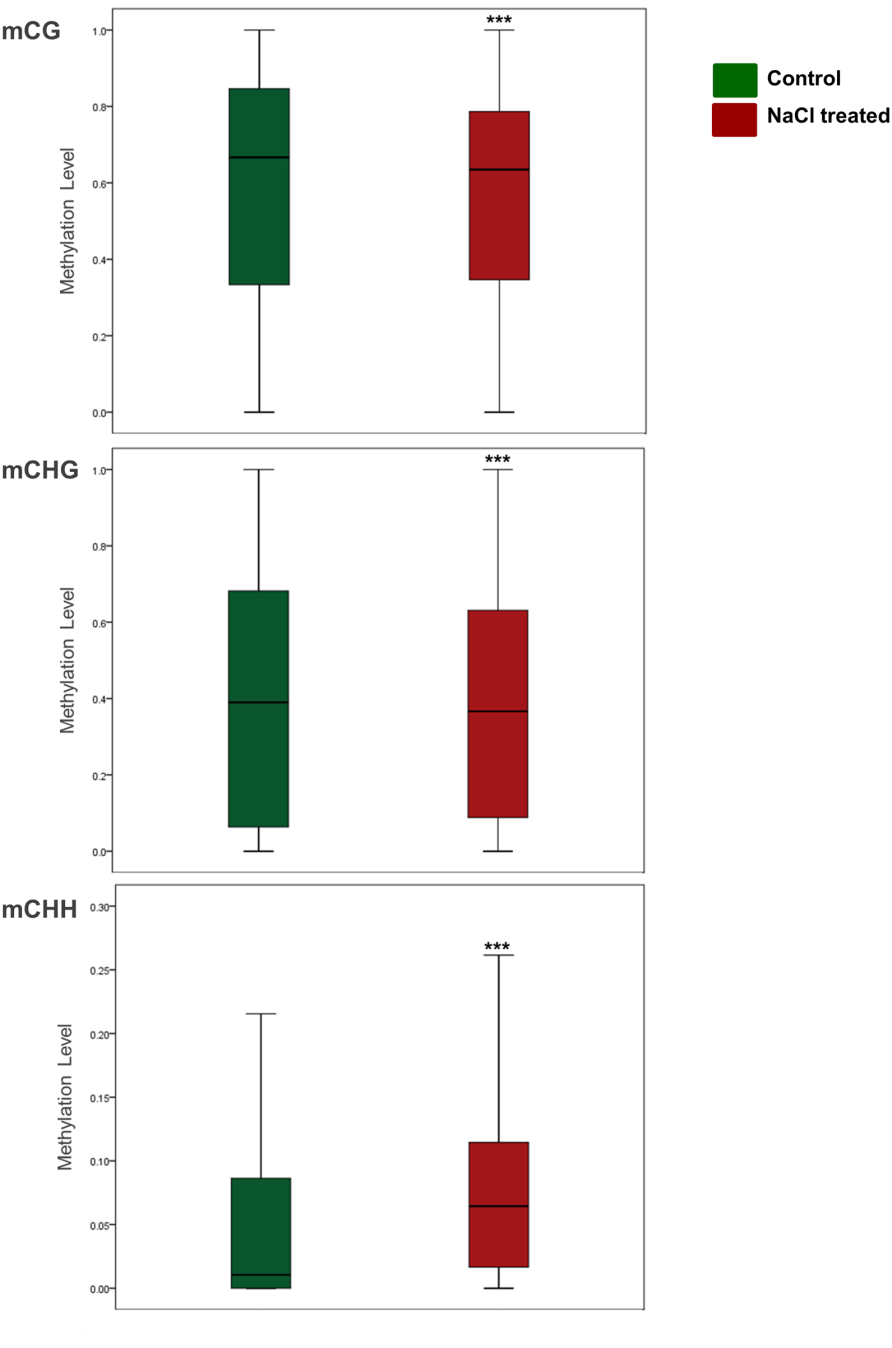
Box plots display the overall methylation level of DMRs (*p* < 0.05) under control and salinity stress conditions (*p* < 0.05 considered to indicate statistical significance. *p < 0.05, **p < 0.01, ***p < 0.001, independent sample t test (two-tailed) was conducted to compare the means of two samples). Bars represent the median.

In order to determine the effect of salinity treatment on the DNA methylation status, a pairwise comparison of the DMRs was carried out. The analysis showed that 2,130,787 DMRs were significantly (*p* < 0.05) altered in response to salinity stress (Table 2). Statistical analysis indicated that the DMRs resulted from CHH methylation were more frequent in date palm genome (46.93%) comparing with CG (27.84%) and CHG methylation (25.23%). Using the methylation status in the DNA extracted from the control plants as a reference, the WGBS analysis showed that there were around 55.84% and 44.16% significantly (*p* < 0.05) hypermethylated and hypomethylated regions, respectively, in DNA extracted from plants exposed to salinity stress (Table 2). Collectively, statistical analysis of the DMRs resulted from three sequence contexts methylation showed a significant (*p* < 0.05) increase of about 24.5% in the net methylation level of the DMRs in the genome upon exposure to salinity. It was noticed that the percentages of hypermethylated DMRs were higher than hypomethylated counterparts in the case of mCHG and mCHH, but this situation was the opposite in the case of the mCG context, where the percentages of the hypermethylated DMRs were less than their hypomethylated counterparts. This implies that DNA methylation at the mCG region decreased when plants were exposed to salinity, while it increased at the mCHG and mCHH regions due to the same stress (Table 2) (Fig 3).

**Table 2:** DMRs obtained from the WGBS analysis. The DMRs with a significant abundance value (*p* < 0.05) in response to salinity are showed.

Methylation context	Hypermethylated DMRs	Hypomethylated DMRs	Total significant DMRs	Methylation changes at DMRs %
mCG	282,712	310,540	593,252	‒4.69
mCHG	277,356	260,186	537,542	3.19
mCHH	629,765	370,228	999,993	25.95
Net methylation change				24.46

### Cluster analysis of the top 100 methylated sites

In order to clearly determine the effect of salinity on the status of DNA methylation, the DMSs that showed significant (*p* < 0.05) changes in methylation levels and had the highest methylation difference between the DNA samples (top 100 DMSs) in the mCG, mCHG and mCHH sequence contexts, regardless of their location within the genome, were selected for hierarchically clustering analysis. The resulted heat maps showed some variations in the level of DNA methylation between the two genomes at the mCG DMSs. However, these variations were enormous in the mCHG and mCHH sequence contexts (S1 Fig). This profound variation indicates the important effect of salinity treatment in modulating DNA methylation.

### The level mCG methylation in the promoter and gene body regions

**Fig 4:**
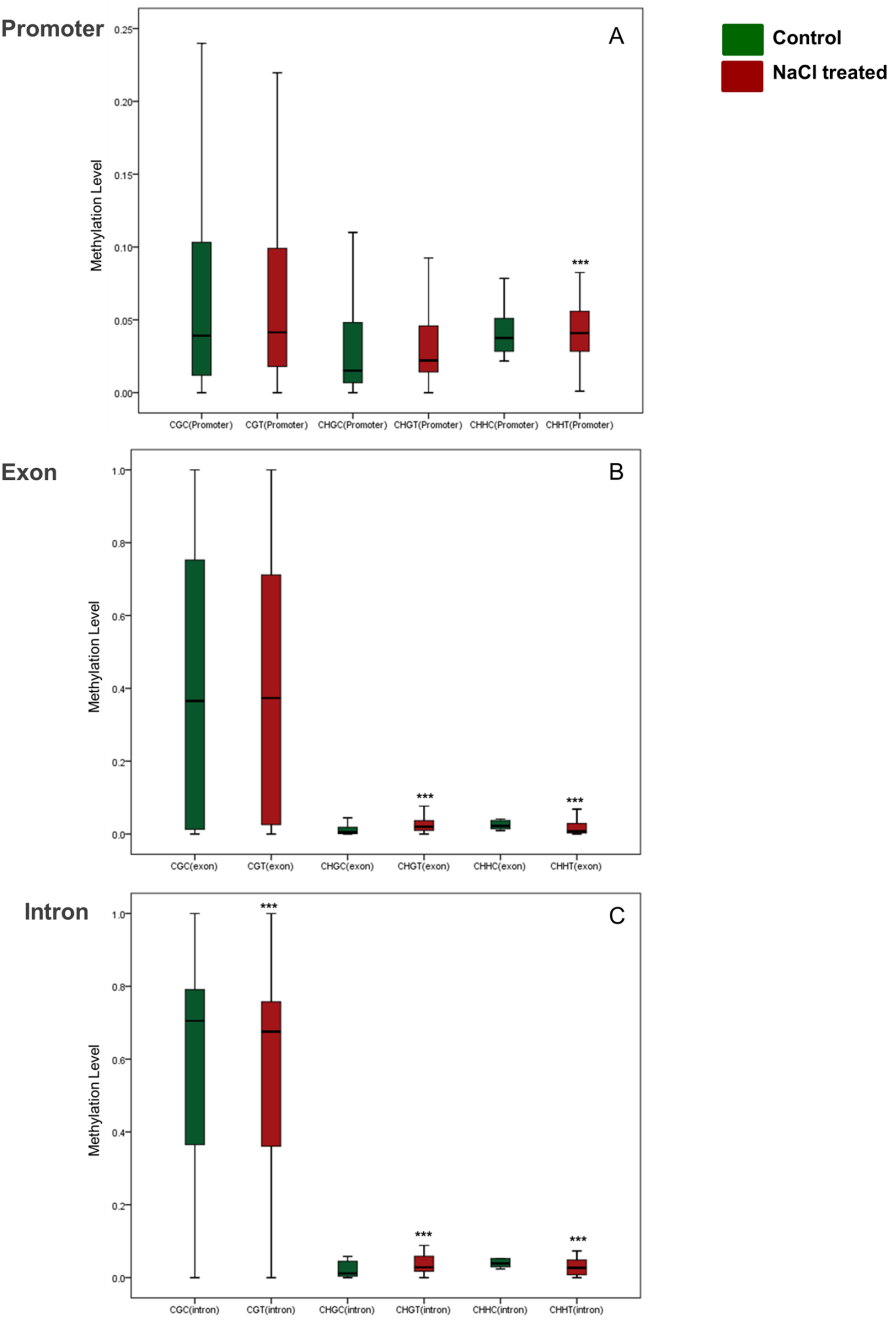
Box plots display the overall methylation level of DMRs (*p* < 0.01,) among promoter (A), exon (B) and intron (C) regions, under control and salinity stress conditions (p < 0.01 considered to indicate statistical significance. *p < 0.05, **p < 0.01, ***p < 0.001, independent sample t test (two-tailed) was conducted to compare the means of two samples). Bars represent the median.

Fortunately, functionally annotated genes and their promoters were significantly covered in this analysis. DNA methylation percentage within the promoter, exon and intron regions of the annotated genes was estimated in the DNA extracted from treated and untreated roots. The DMRs that showed a significant methylation variation (*p <* 0.01) in the studied regions in response to salinity stress was used in the analysis. The results showed that the exonic DMRs of mCG methylation had higher methylation level than the promoter’ DMRs, regardless of the salt stress treatment (Fig 4A and 4B). Generally, there was an increase in the methylation level of DMRs located within promoters after exposure to salinity stress (Fig 4A). This trend was also noticed with exonic DMRs except for these related to mCHH sequence context (*p <* 0.01) (Fig 4B). However, the intronic DMRs of mCG and mCHH methylation showed a reduced (*p <* 0.01) DNA methylation level due to the salt treatment. Contradictory, the methylation level of the intronic DMRs of mCHG was increased (*p <* 0.01) due to the same treatment (Fig 4C).

### DNA methylation landscape across the genes and the flanking regions

**Fig 5:**
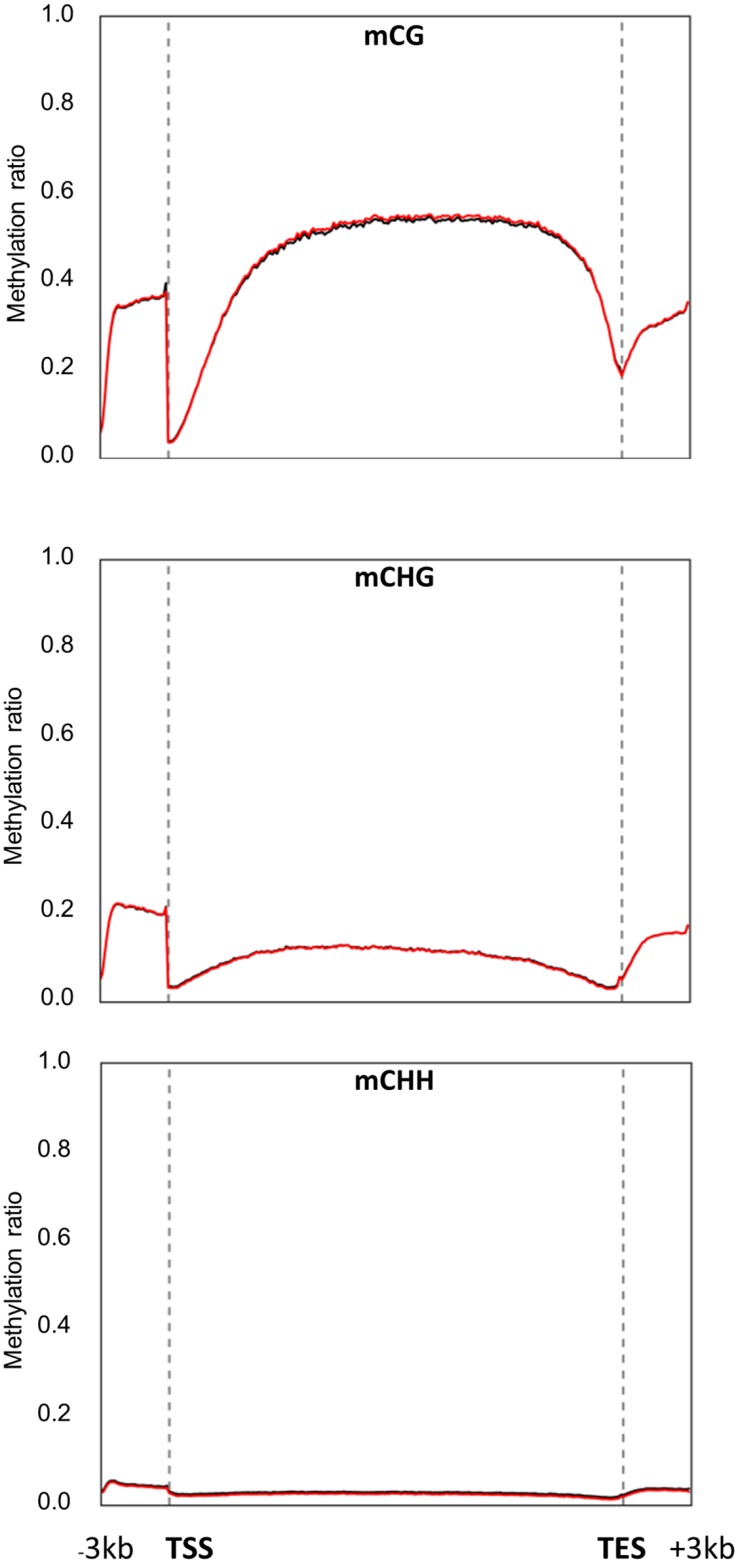
DNA methylation overview across the gene and the flanking regions based on the distribution of mCG, mCHG and mCHH differentially methylated sites (*p* < 0.001, *FDR* ≤ 0.05) under control (red lines) and salinity stress (black lines) conditions. These sites were plotted against gene structural features, including the promoter, gene body and flanking regions, which include transcription start site (TSS), transcription end site (TES), and 3Kb up- and down-stream regions.

In order to determine the landscape of the DMSs within the annotated genes and their flanking regions, and also to study the dynamics of DNA methylation in response to salinity stress in these regions, an overview of the global DNA methylation pattern was constructed based on the distribution of the mCG, mCHG and mCHH DMSs across the defined gene features that are available in the genome database. This includes the protein-coding region, the transcription start site (TSS), the transcription end site (TES) and their 3Kb flanking regions (Fig 5).

The results revealed that the DNA methylation patterns among the mCG level were relatively low (~ 35%) in the ‒3Kb upstream the TSS regions; however, this level was dramatically dropped to the lowest level in the TSS regions, then gradually increased to nearly a constant level (~ 50%) in the coding region. This methylation level decreased again toward the TES regions before it increased again in the downstream +3Kb regions (Fig 4A). The methylation level in the mCHG sequence context was the highest (~ 20%) among the other regions in the promoter sequences and decreased close to the TSS, but this level slightly increased in the coding regions and decreased again near the TES region. Subsequently, this DNA methylation level gradually increased in the +3Kb flanking regions (Fig 5B). A similar trend of DNA methylation was also observed at the mCHH DMSs (Fig 5C); however, the DNA methylation level was too low (~ 3% at most) in the different gene regions. Collectively, salinity treatment did not show a clear effect on DNA methylation levels at the 5mdCs located within the gene regions.

**Fig 6:**
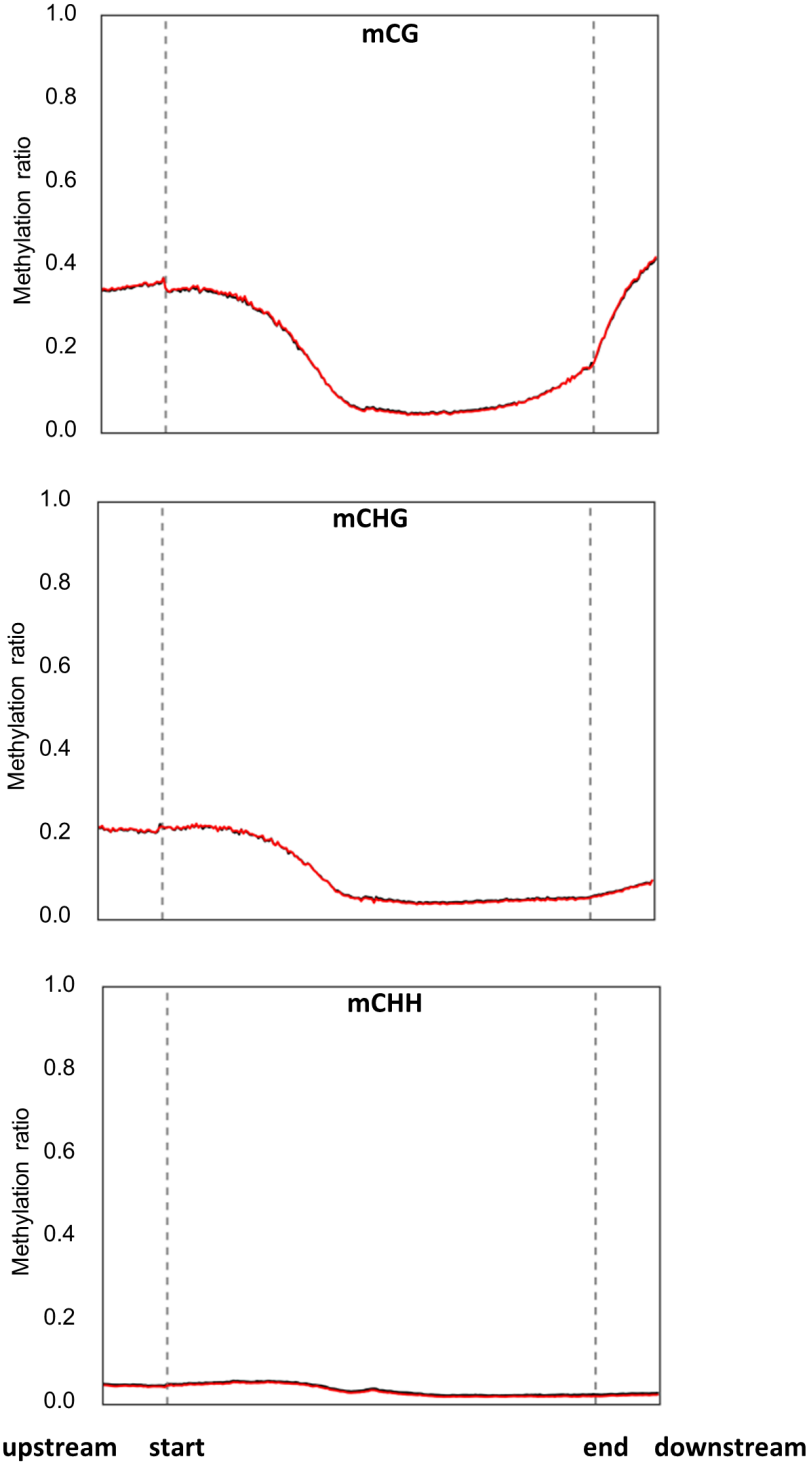
DNA methylation overview across the promoter and the flanking regions based on distribution of mCG, mCHG and mCHH differentially methylated sites (*p* < 0.001, *FDR* ≤ 0.05) under control (red lines) and salinity stress (black lines) conditions. These sites were plotted against promoter sequences and flanking regions, which include 1Kb up- and down-stream promoter region.

The methylation landscape was also described for the annotated promoters and their flanking regions (Fig 6).

mCG showed the highest level of DNA methylation in the start regions of the promoters as compared to the other sequence contexts; however, it was observed that the DNA methylation of mCG and mCHG was dramatically decreased toward the end of the promoters (Fig 6). It was not possible to perceive obvious differences in DNA methylation profiles between the DNA extracted from the treated and untreated plants using this method.

### Mass spectrometry and ELISA revealed an increase in the global genomic 5mdcs in response to salinity

The mass spectrometry analysis revealed undetectable amounts of 5-hydroxymethyl-2’-deoxycytidine (5hmdC). However, quantification of global 5mdC revealed the presence of 5.23 ± 0.08% (mean ± SD) and 7.88 ± 0.90% (mean ± SD) of 5mdC of the total cytosines of DNA samples extracted from the control and NaCl-treated plants, respectively. Statistical analysis revealed that these amounts account for a significant (*p* = 0.007) increase of 50.7% in global DNA methylation occurring in the genomes of the salinity-treated plants. Similarly, ELISA showed a relatively significant (*p* = 0.05) increase of 37.2% in the global 5-mCs of the DNA samples extracted from roots of plants grown in saline conditions.

### DMSs profiles across the genes and their flanking regions showed distinct influence on gene expression

Sequencing of the mRNA purified from roots revealed a total number of 59,330,361 and 62,543,394 reads sequenced from the salinity-treated and untreated tissues, respectively (Table 3). The genome coverage was about 88% and 89% for the control and the salt treated samples, respectively. These reads were combined into 370,538 contigs, with a sequence length varied from 21 to 168,337 bp. The analysis indicated the presence of around 6,405 DEGs in response to salinity (S1 Table), such that 64% and 36% of these genes were significantly (*p* < 0.001, *FDR* ≤ 0.05) upregulated and downregulated, respectively.

**Table 3:** Re-sequencing results of total mRNA isolated from NaCl-treated and control root tissues.

Sample	Number of reads	Total (bp)	Total Length of contigs	Total number of contigs	Maximum length (bp)	Minimum length (bp)	DEG (≥2 fold change, *p*-value, FDR < 0.05)	
Control	62,543,394	3,127,169,700	297,394,902	370,538	168,337	21	Up-regulated	4,093
NaCl-Treated	59,330,361	2,966,518,050					Down-regulated	2,312

**Fig 7:**
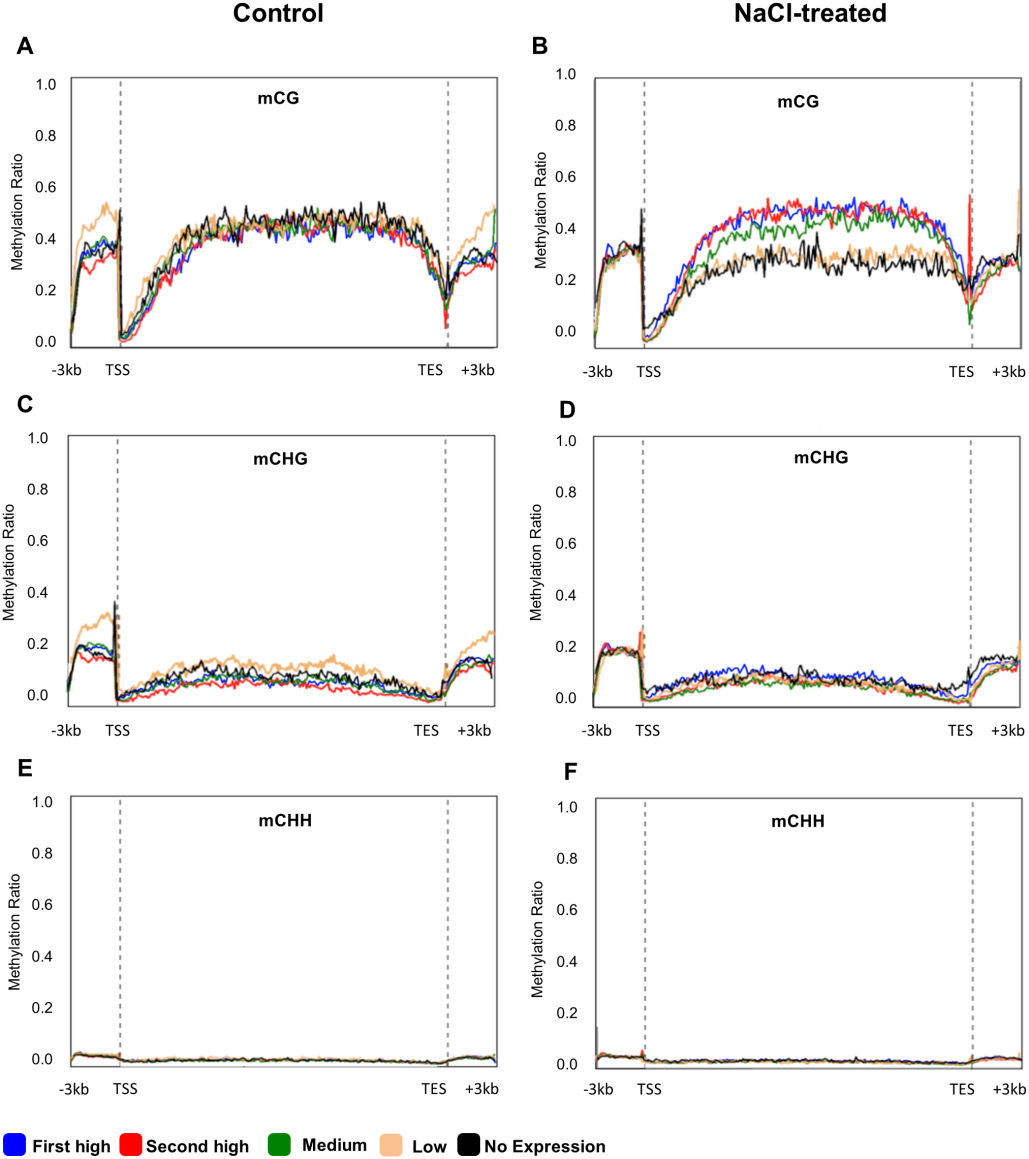
DNA methylation (mCG, mCHG and mCHH) profile among DEGs (*p* < 0.001, *FDR* ≤ 0.05), grouped based on their expression level (first high (blue line), second high (red line), medium (green line), low (beige line) and no expression (black line)). The Y-axis represents the DNA methylation ratio of each group, while the X-axis represents studied gene regions of DMGs including protein-coding region, the transcription start site (TSS), the transcription end site (TES) and 3Kb up and down-steam the TSS and the TES, respectively.

**Fig 8:**
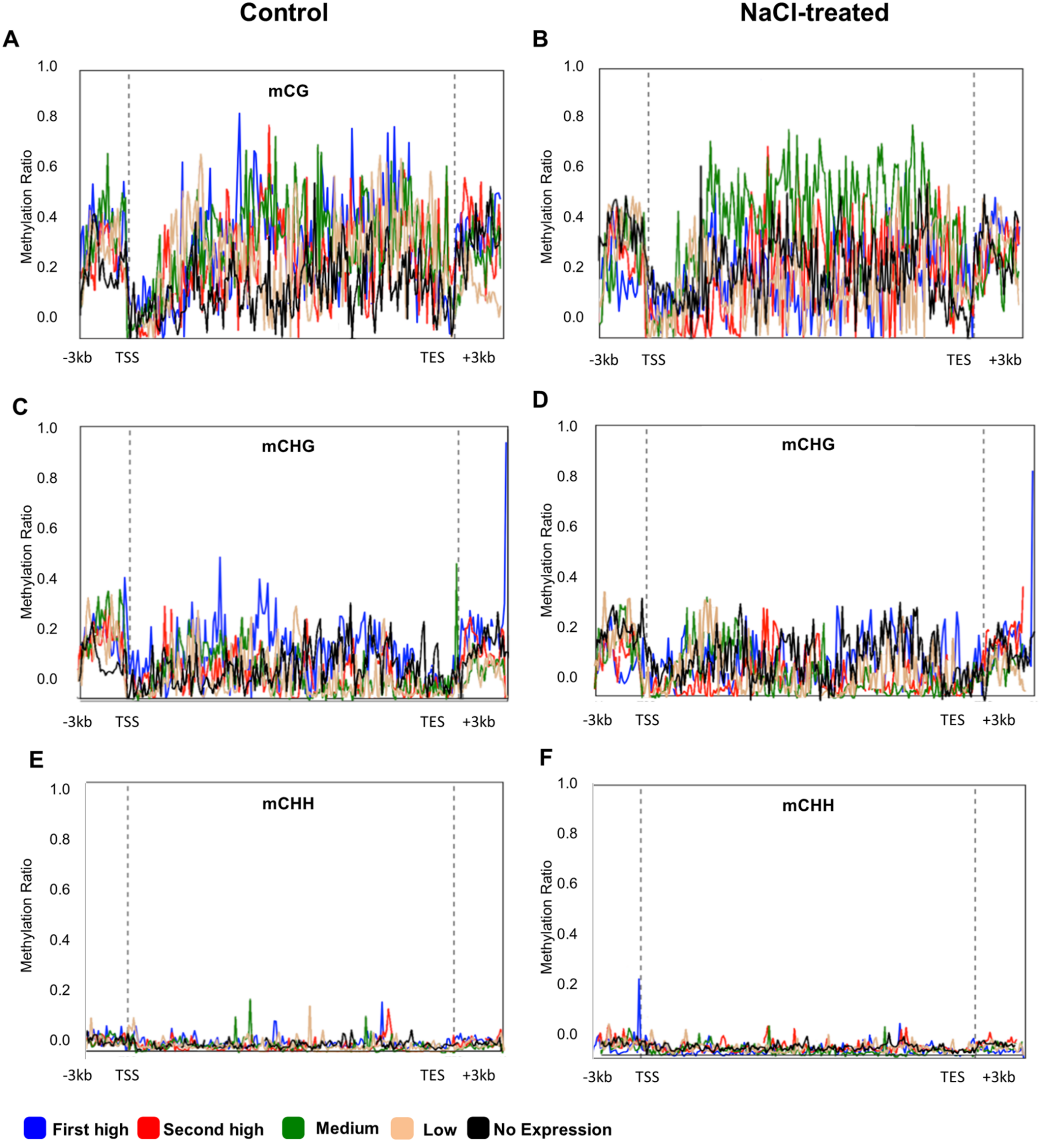
DNA methylation (mCG, mCHG and mCHH) profile among pseudogenes, grouped based on their expression level (first high (blue line), second high (red line), medium (green line), low (beige line) and no expression (black line)). The Y-axis represents the DNA methylation ratio of each group, while the X-axis represents studied gene regions of DMGs including protein-coding region, the transcription start site (TSS), the transcription end site (TES) and 3Kb up and down-steam the TSS and the TES, respectively.

In order to investigate the relationship between DNA methylation and gene expression, the DEGs under salinity treatment were classified into five different groups based on their expression value (first high, second high, medium, lower, and no expression). Subsequently, DNA methylation profiles at the mCG, mCHG and mCHH sites within the functional (protein coding) genes (Fig 7) and pseudogenes (Fig 8) were correlated with the corresponding gene expression value of each group. Under the control conditions, the results showed that there was a negative correlation between the level of mCG DNA methylation within the ‒3Kb region and gene expression levels. This relationship was also found in the +3kb region (Fig 7A).

On the other hand, under salinity conditions, high gene expression was associated with high mCG methylation levels, especially when it occurred in the gene body regions (Fig 7B). However, when plants grew under control conditions, DNA methylation at the mCHG DMSs located within all regions was associated with low gene expression (Fig 7C). When plants were exposed to salinity, a slight level of methylation within the coding regions was associated with gene expression (Fig 7D). The results also showed that there was unclear effect of the DNA methylation level at the mCHH sites on gene expression (Fig 7D). Irrespective of the environmental conditions, a clear negative relationship was observed between 5mdC methylation levels around TSS and gene repression levels (Fig 7). However, mCG methylation around TES was associated with gene activation only when plants were exposed to salinity (Fig 7B).

In order to compare the effect of DNA methylation on the functional gene and its effect on the pseudogenes in terms of transcriptome abundance, DNA methylation levels of the three sequence contexts were plotted against the different groups of pseudogenes classified based on their expression level, as described above (Fig 8). In general, the DNA methylation levels within the pseudogene regions were relatively higher than the levels found within the functional genes (Figs 7 and 8). For example, the highest level of mCG methylation observed in the pseudogenes was around 80%, while the highest level in the coding genes was around 50%. The results also showed that DNA methylation in the mCG and mCHG sequence contexts was positively correlated to gene expression over most of the studied regions when plants were grown under the control conditions (Fig 8A and 8C); the first high group of differentially expressed pseudogenes (blue lines) has the highest methylation ratio of mCHG sequence context. However, when plants were treated with salt, the level of DNA methylation was maintained, but it was mainly associated with a lower level of pseudogene expression (Fig 8B and 8D). As an example, the third group of expression level (green lines) showed the highest level of mCG methylation. Profiling of mCHH among pseudogene expression groups did not show a clear effect on gene expression, irrespective of the growth conditions of the plants (Fig 8E and 8F).

### The effect of DMGs on gene expression

Among 6,405 DEGs (*p* < 0.001, FDR ≤ 0.05), 71.6% contained mCG, 79.7% contained mCHG, and 94.2% contained mCHH sites (S2 Fig). These genes were considered to be DMGs, and were recognized based on pairwise differential comparison using the t-test (*p* ≤ 0.05). In order to determine the effect of different methylated regions within the genes (promoter, exon, and intron) on gene expression under control and salinity conditions, a statistical correlation between DMGs and their gene expression values was studied using the measure of Pearson’s linear correlation. Specifically, the correlation was created between the log_2_ of mRNA expression (FPKM values) and the log_2_ of the methylation ratio. It was observed that the impact of DNA methylation on gene expression was modulated by the environmental conditions, in which there was generally an opposite effect of the DNA methylation on gene expression as a result of the changes in the environmental conditions (control versus salinity).

The results revealed that there was an insignificant negative correlation between mCG methylation levels located within the promoters and gene expression when plants were grown under both control and salinity conditions. However, there was a significant (*p* ≤ 0.05) positive correlation between the methylation level at the mCHG and mCHH sites located within the promoters and gene expression when plants were grown under control conditions. On the contrary, when plants were grown under salinity conditions, this relationship was significantly (*p* ≤ 0.01) negative (Table 4).

**Table 4:** Correlation between DMGs and gene expression based on the Pearson’s linear correlation. Asterisk stands for statistical significance (* if *p* ≤ 0.05 and ** if *p* ≤ 0.01).

Genomic Region (mCG)	Control		NaCl‒treated	
	Correlation Coefficient (*r*)	*p*‒value	Correlation Coefficient (*r*)	*p*‒value
Promoter	‒0.054	0.348	‒0.053	0.359
Exon	‒0.009	0.826	0.011	0.788
Intron	‒0.085**	0.008	0.048	0.131
All regions	‒0.114**	1.00E^‒13^	0.064**	6.75E^‒06^
**Genomic Region** (mCHG)				
Promoter	0.106*	0.049	‒0.260**	9.96E^‒07^
Exon	0.140**	2.70E^‒04^	‒0.258**	1.10E^‒11^
Intron	0.077**	0.004	‒0.153**	1.02E^‒08^
All regions	0.126**	1.00E^‒13^	‒0.184**	1.00E^‒13^
**Genomic Region** (mCHH)				
Promoter	0.246**	2.00E^‒06^	‒0.313**	7.67E^‒10^
Exon	0.137**	3.25E^‒04^	‒0.162**	2.20E^‒05^
Intron	0.105**	5.10E^‒05^	‒0.187**	5.75E^‒13^
All regions	0.143**	1.00E^‒13^	‒0.206**	1.00E^‒13^

The methylation profile of the mCG sequence contexts located within the exon regions of the DMGs showed a trend of a negative correlation with the gene expression when plants were grown under control conditions, but this correlation was reversed when the plants were grown under salinity conditions. The correlation between the methylation level of the mCHG and mCHH located within the exons and the gene expression was significantly (*p* ≤ 0.01) positive when plants were grown under control conditions; however, this relationship was significantly (*p* ≤ 0.01) negative when plants were exposed to salinity.

The methylation profile of the mCG sequence contexts located within the intron regions of the DMGs showed a significant (*p* ≤ 0.01) negative correlation with the gene expression when plants were grown under control conditions, but this relationship was insignificantly positive when the plants were grown under salinity conditions. The correlation between the methylation levels of the mCHG and mCHH encountered within the introns and the gene expression was significantly (*p* ≤ 0.01) positive when plants were grown under control conditions; however, this relationship was significantly (*p* ≤ 0.01) negative when plants were exposed to salinity.

When the methylation profiles of the whole gene regions were correlated with their gene expression levels, the statistical analysis showed that there was a significant (*p* ≤ 0.01) negative relationship between the DNA methylation levels of mCG and gene expression when plants were grown under control conditions. The same negative relationship was found in mCHG and mCHH when plants were exposed to salt stress (Table 4). On the other hand, when plants were exposed salt stress, there was a significant (*p* ≤ 0.01) positive correlation between the levels of DNA methylation at the mCG sites and gene expression. The same positive relationships were observed between the methylation levels of mCHG and mCHH and gene expression when plants were grown under control conditions.

### Functional annotation of DMGs

In order to obtain a comprehensive idea of the biological functions of the DMGs in the date palm, the protein-coding DNA sequence of the DMGs from the three sequence contexts were separately annotated and designated gene ontologies (GOs).

Each sequence context was separately analyzed based on three GOs: biological process (BP), molecular function, and cellular component (S3–S5 Figs). The functional annotation of the DMGs included one of the three sequence contexts: mCG (S3 Fig, S2 Table), mCHG (S4 Fig, S3 Table) and mCHH (S5 Fig, S4 Table). The results showed that the DMGs were involved in the biological processes of oxidation-reduction, regulation of transcription, protein phosphorylation, proteolysis, transport and methylation. The DMGs were found to be also involved in the integral components of the membrane, nucleus, mitochondrion, cytoplasm, chloroplast, plastids, plasma membrane and cytosolic cellular components. These genes were also encoded for molecular functions, including the binding of proteins, ATP, DNA, ions, cations and nucleic acids.

**Fig 9:**
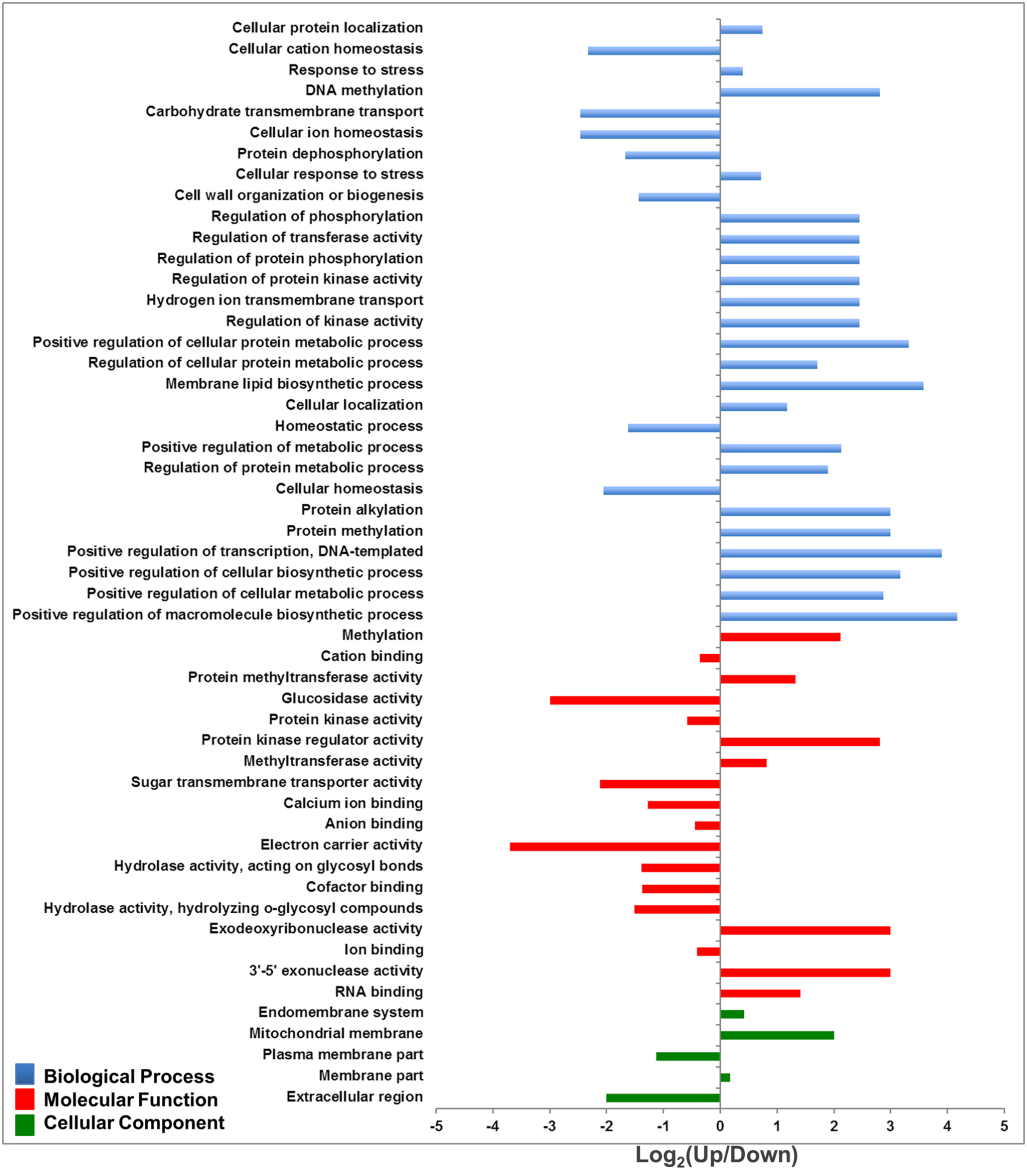
Functional enrichment analysis of significant DMGs (*p* ≤ 0.05) including mCG based on the categories biological process, cellular component and molecular function. Fisher’s exact test analysis considered the upregulated DMGs as a test group.

Differential enrichment analysis based on Fisher’s exact test (*p* < 0.05) was carried out in order to identify the differential presented GOs. The analysis showed that downregulated genes containing mCG were enriched in salinity-responsive categories, such as cellular ion and cation homeostasis, carbohydrates transmembrane transport, cation and anion binding, protein kinase activity, protein dephosphorylation and ion binding. These categories were also enriched with GOs acting on plasma membranes and extracellular regions (Fig 9, S5 Table).

Upregulated genes containing mCHG were enriched with DNA and protein methylation, vesicle-mediated transport, intracellular transport, hydrogen and proton transport, vacuole organization, protein transporter, N-methyltransferase, 3'-5' exonuclease. These GOs were found to be involved in the whole membrane, the bounding membrane of organelles, endomembrane systems and chloroplast parts (S4 and S6 Figs, S6 Table). The upregulated genes containing mCHH were abundantly enriched with cellular potassium ion transport, vacuolar transport, DNA methylation, amino acid activation, protein, proton, and hydrogen ion transmembrane transport, solute:cation and solute: proton antiporter activity, hydrogen ion transmembrane transporter activity, protein transporter activity, 3'-5'-exodeoxyribonuclease activity and monovalent inorganic cation transmembrane transporter activity (S5 and S7 Figs, S7 Table).

Functional annotation analysis of the DMGs also revealed that the DMGs encoded 620 enzymes. The list included 199 DMGs that embraced mCG sites, 206 that embraced mCHG sites, and 215 that embraced mCHH sites. These enzymes were mapped within 318 unique pathways (S8–S10 Tables). This includes those pathways that are involved in cysteine and methionine metabolic pathway (S8 Fig).

## Discussion

DNA methylation is an epigenetic mechanism that regulates key cellular processes in eukaryotes [11–13, 47]. Recent studies that focused on methylome alterations in response to abiotic stresses have shown variable patterns of DNA methylation among different plant species. However, these patterns are inconsistent, especially in perennial species [23]. Changes in DNA methylation are usually associated with alterations in plant growth and development [22]. In the current study, salinity showed a negative effect on the growth of the leaves and roots of the treated seedling. An abnormal development of the leaves was also associated with a reduction in photosynthetic performance. This result is consistent with previously published research on date palms in which transcriptome analysis showed a negative effect of salinity on the expression of some key genes associated with photosynthesis [48, 49]. These changes in gene expression could be due to alterations in DNA methylation in the leaf tissues.

Root systems represent the very first line of defense against salinity stress damage. Therefore, epigenetic alterations associated with physiological changes are important sources of information needed to decode the salinity tolerance mechanisms in plants. Impaired root growth and development in date palms due to salinity could be the result of alterations in DNA methylation, because it was previously documented that changes in DNA methylation caused abnormal root development in Arabidopsis [50].

In this project, a high throughput analysis of single-base resolution DNA methylome as well as transcriptome analysis was used to investigate the regulation of salinity-responsive genes by DNA methylation in date palm roots. WGBS is the most versatile method used to obtain a comprehensive idea about DNA methylation, especially for studies aiming at constructing the first methylome map of an organism such as the date palm [51].

In this project, DNA of each sample was extracted from a pool of root tissues collected from eight different plants. Using of this pool of plant tissues in the WGBS analysis can minimize the epigenetic variations, which may occur between the individual seedlings of the same treatment. Each pool of these DNA was considered as a biological sample. Obviously, including more experimental and experimental replicates in the WGBS analysis may improve the quality of the results however, WGBS sequencing is expensive and few laboratories can afford it. Although we consider the results obtained from this project as the first draft methylome map for the date palm, these results are novel and largely consistent with the known facts about methylome and transcriptome relationships.

The unique reads obtained by bisulfite-converted sequencing of the control and the salinity-treated samples had a mapping efficiency of around 41%. This value is consistent with the range of the typical mapping efficiency of bisulfite-converted DNA, which is 30–50% [52]. In addition, our methylome sequencing analysis results have an average read depth of 17X per each DNA strand (34X total coverage), a coverage which was previously recommended by the National Institutes of Health (NIH) Roadmap Epigenomics Project as sufficient [53]. The results presented in this study were obtained despite the fact that the date palm genome is not yet completely sequenced or assembled. It is known that the performance of the WGBS technique mainly depends on the quality of the reference genome [53, 54] and the alignment efficiency of the bisulfite-converted sequencing reads to the reference genome [55].

Global quantification of 5-mCs using mass spectrometry showed a similar amount to those previously found in other plant species, which ranged from 5% to 30% of total cytosine [56, 57]. Mass spectrometry also showed a substantial increase in the DNA methylation level after exposure to salt stress. Frequently, overmethylation is associated with the salt stress and salinity tolerance phenotype in plants. For example, when Arabidopsis is exposed to salinity, the global DNA methylation level increases [58]. Such an increase was also detected in *Medicago* species [31] and *Mesembryanthemum crystallinum* [59]. Moreover, salt-tolerant wheat cultivar are more methylated than their susceptible counterparts [60]. On the other hand, previously published reports showed that global DNA demethylation is associated with salinity tolerance in plants [61]. Accordingly, the results obtained in this study are not surprising, because the effect of salinity on DNA methylation varies based on plant species, genotype and the organ within the same plant [62–64].

Mass spectrometer analysis could not detect 5HmdC in the date palm genome. This is consistent with the fact that only trace amounts of 5HmdC were detected in Arabidopsis and these amounts could be the result of random oxygen species reactivity with 5-mCs [65]. In mammals, the ten eleven translocation 1 (TET1) family of enzymes oxidizes 5mdC to 5HmdC [9, 66, 67]; however, none of this family of related enzymes were previously reported in plant species. Consequently, 5HmdC was not detected in a remarkable amount [68].

The methylated mCHH was the most abundant type among the total significant DMRs, followed by mCG methylation (Table 2). In fact, the methylation in the mCG sequence context is the most persistent and stable in the genome [8, 46]. The differential cytosine methylation analysis showed that the date palm genome was hypermethylated in response to salt stress at these DMRs of a significant (*p* < 0.05) number of reads (Table 2).

DMRs may include more than one type of methylated sequence contexts thus, the presence of overlapped methylated sequences will affect the calculations regarding the total levels of methylation within DMRs. However, since the number of hypermethylated mCHH within the DMRs is significantly high (26% higher than their hypomethylated counterparts), the chance that the overlapped methylated sequence contexts could change the conclusion that salinity increases global DNA methylation is unlikely especially because this conclusion is supported by mass spectrometry and ELISA analyses.

Gene repression can take place when the transcription factor is unable to access specific motifs located within the promoter regions [69]. An increase in the methylation level within the promoter regions is usually associated with a low or even no gene expression [69]. However, a positive correlation was previously documented between gene body methylation and gene expression [8, 18, 26]. In the date palm, mCG methylation within the promoter was associated with gene repression; however, other types of cysteine methylation located within other gene features showed a salinity stress-dependent gene expression. 5-mC bisulfite sequencing and gene expression analysis in maize seeds revealed the opposite effect of mCG methylation located within the coding regions on transcription activation, as compared to mCHG and mCHH methylation [70]. This is consistent with our results. However, when plants were exposed to salt stress, the mCHG and mCHH methylation levels were inversely correlated with gene expression. Therefore, it was clear from the results of our study that salinity alters the commonly established relationship between DNA methylation and gene expression. This is likely because under stress conditions, there are additional epigenetic factors that may be involved in gene regulation networks, such as sRNA molecules [71, 72].

It was noticed from the results that the DNA methylation level of pseudogenes was higher than those found in the protein-coding genes. This is consistent with the previous findings of the Arabidopsis methylome [13, 73]. There were some differences between the effect of DNA methylation on gene expression of the functional genes and of that of the pseudogenes. DNA methylation in the pseudogenes had a lower impact on the expression level it did in the case of the coding genes. Recently, it was found that pseudogene expression plays a crucial role in regulating the expression of their parental genes and other unrelated genes; however, the underlying mechanism is unclear [74].

Under both control and salt stress conditions, our analysis indicated that mCG and mCHG methylation around the TSS has negatively affected gene expression. However, mCG methylation around the TES was positively correlated with gene expression when plants were exposed to salinity. It was previously demonstrated that mCG methylation around the TSS and TES have a negative effect on gene expression in rice [75] and maize [70].

GO enrichment analysis showed that the upregulated DMGs of the three sequence contexts were abundantly involved in DNA methylation. This is could be evidence of an increased methylation level under salinity stress. In addition, most of the transporter and antiporter coding proteins involved were in abundantly enriched categories in the downregulated DMGs containing mCG. However, these salinity response-associated ontologies were abundantly enriched in the upregulated DMGs containing mCHG and mCHH. This finding is in agreement with the transcriptome/methylome analysis based on Pearson’s correlation, which showed that under salinity stress there was a negative correlation between the mCG methylation ratio and expression level, while there was a positive correlation between mCHG and mCHH methylation ratios and expression levels.

Mapping DMGs on the KEGG maps revealed that the cysteine and methionine metabolic pathway was enriched with enzymes coded by methylated genes in three sequence contexts. Interestingly, it was previously observed that this pathway is actively involved in abiotic stress response [76]. Sulfur-containing amino acids (cysteine and methionine) are sensitive to ROS-mediated oxidation. For example, oxidation changes methionine to S- and R-methionine sulfoxide diastereoisomers. Two types of methionine sulfoxide reductases, MSRA and MSRB, can specifically reverse this reaction. Based on functional characterization analysis in rice, overexpression of the gene coding MSRA (*OsMSRA4*.*1)* showed an enhanced growth under salt stress[76].

In conclusion, this initial study identified widespread salinity-induced changes in 5mC methylation and transcriptome abundance in the roots of the date palm. The methylation changes that occurred within various regions of the gene showed an impact on gene expression. While the DNA methylation demonstrated the common regulation pattern known in eukaryotes on gene expression when plants grow under optimum environmental conditions, this study revealed that when plants grow under salinity stress, DNA methylation is not the primary agent that controls gene expression in date palm roots. The results obtained from this study could highlight the importance of DNA methylation in epigenetic remodeling under stress conditions, and the information encoded by the methylome could be beneficial for crop improvement strategies.

## Supporting information

S1 Fig Clustering heatmap analysis of the top 100 differentially methylated sites (DMSs) (*p* < 0.05). Dendogram of hierarchical clustering was obtained based on DNA methylation ratio of mCG, mCHG and mCHH sequence contexts. Red and yellow color scale represents individual sites that are 0% and 100% methylated, respectively.
(TIF)

S2 Fig Classification of the differentially expressed genes (DEGs) (6,405 genes) which were recognized as differentially methylated genes (DMGs) in response to salinity stress based on their methylation status. (TIF)

S3 Fig Gene ontology (GO) analysis of DMGs for each sequence context mCG of date palm epigenome in response to salinity stress, based on three sets of ontologies: (A), molecular function (B), and cellular component (C) against the GO databases. (TIF)

S4 Fig Gene ontology analysis of DMGs for each sequence contexts mCHG of date palm epigenome in response to salinity stress, based on three sets of ontologies; (A), molecular function (B), and cellular component (C) against the GO databases. (TIF)

S5 Fig Gene ontology analysis of DMGs for each sequence contexts mCHH of date palm epigenome in response to salinity stress, based on three sets of ontologies; (A), molecular function (B), and cellular component (C) against the GO databases. (TIF)

S6 Fig Functional enrichment analysis of significant DMGs (*p* ≤ 0.05) including mCHG based on the categories biological process, cellular component and molecular function. Fisher’s exact test analysis considered the upregulated DMGs as a test group.
(TIF)

S7 Fig Functional enrichment analysis of significant DMGs (*p* ≤ 0.05) including mCHH based on the categories biological process, cellular component and molecular function. Fisher’s exact test analysis considered the upregulated DMGs as a test group.
(TIF)

S8 Fig Schematic representation of enzymes coded by mCHG DMGs. These enzymes might function in the cysteine and methionine metabolic pathway.
(TIF)

S1 Table Differentially expressed genes (DEGs) (*p* < 0.001, *FDR* ≤ 0.05) under salinity stress. (XLSX)

S2 Table Functional annotation and gene ontology of DMGs for the mCG sequence context. (XLSX)

S3 Table Functional annotation and gene ontology of DMGs for the mCHG sequence context. (XLSX)

S4 Table Functional annotation and gene ontology of DMGs for the mCHH sequence context. (XLSX)

S5 Table Functional enrichment analysis of significant DMGs (*p* ≤ 0.05) for the mCG sequence context. Fisher’s exact test analysis considered the upregulated DMGs as a test group.
(XLSX)

S6 Table Functional enrichment analysis of significant DMGs (*p* ≤ 0.05) for the mCHG sequence context. Fisher’s exact test analysis considered the upregulated DMGs as a test group.
(XLSX)

S7 Table Functional enrichment analysis of significant DMGs (*p* ≤ 0.05) for the mCHH sequence context. Fisher’s exact test analysis considered the upregulated DMGs as a test group.
(XLSX)

S8 Table Mapping of DMGs for the mCG sequence context on the metabolic pathways based on annotated coding enzymes. (XLSX)

S9 Table Mapping of DMGs for the mCHG sequence context on the metabolic pathways based on annotated coding enzymes. (XLSX)

S10 Table Mapping of DMGs for the mCHH sequence context on the metabolic pathways based on annotated coding enzymes. (XLSX)
