# Supplementary figures and images for: Differential DNA methylation and transcription profiles in date palm roots exposed to salinity
Source: PLoS One. 2018 Jan 19;13(1):e0191492. doi: 10.1371/journal.pone.0191492 (PMC5774813; doi:10.1371/journal.pone.0191492)

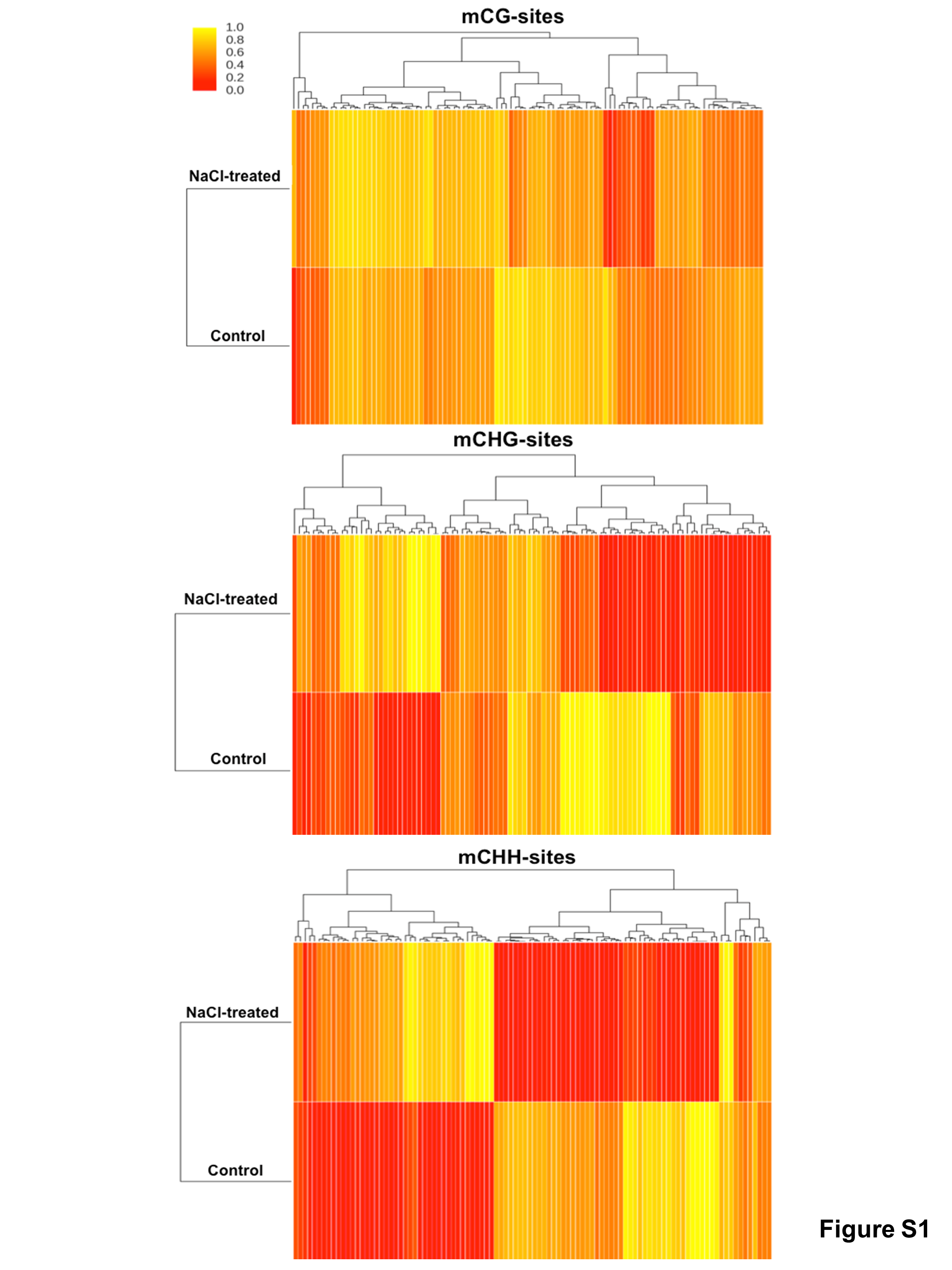

Supplement: S1 Fig — Dendogram of hierarchical clustering was obtained based on DNA methylation ratio of mCG, mCHG and mCHH sequence contexts. Red and yellow color scale represents individual sites that are 0% and 100% methylated, respectively. (TIF) [file pone.0191492.s001.TIF]

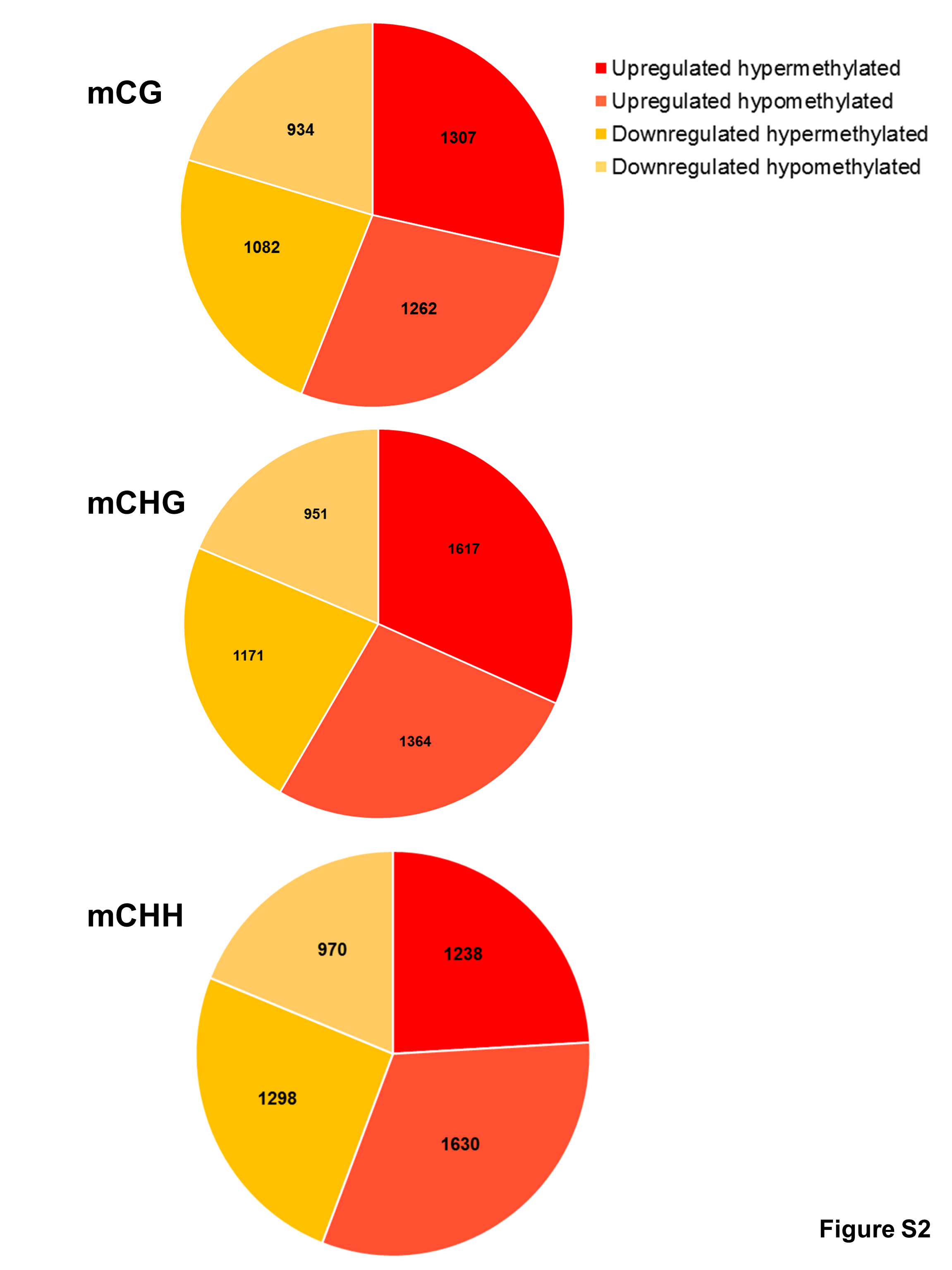

Supplement: S2 Fig — (TIF) [file pone.0191492.s002.TIF]

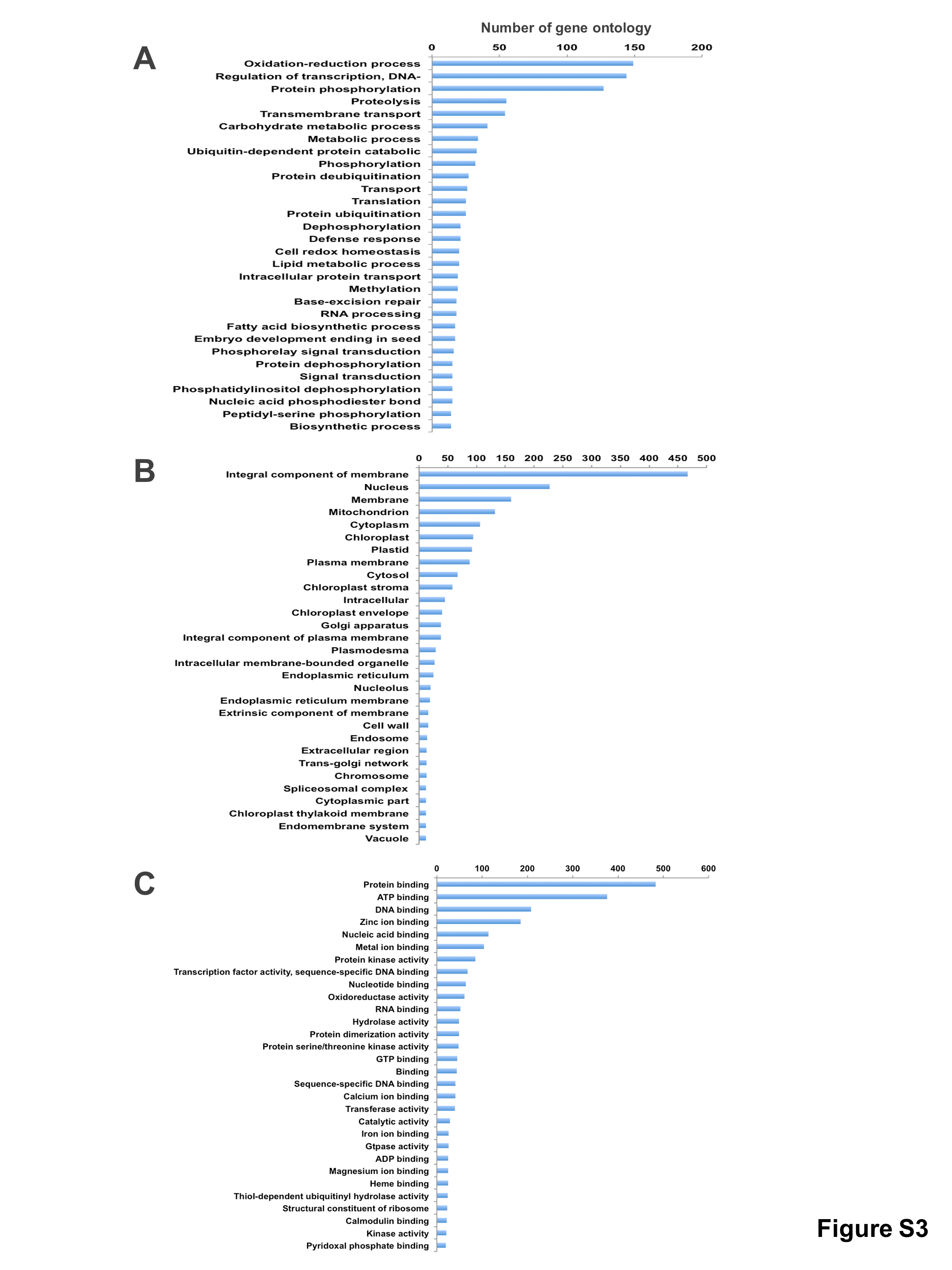

Supplement: S3 Fig — (TIF) [file pone.0191492.s003.TIF]

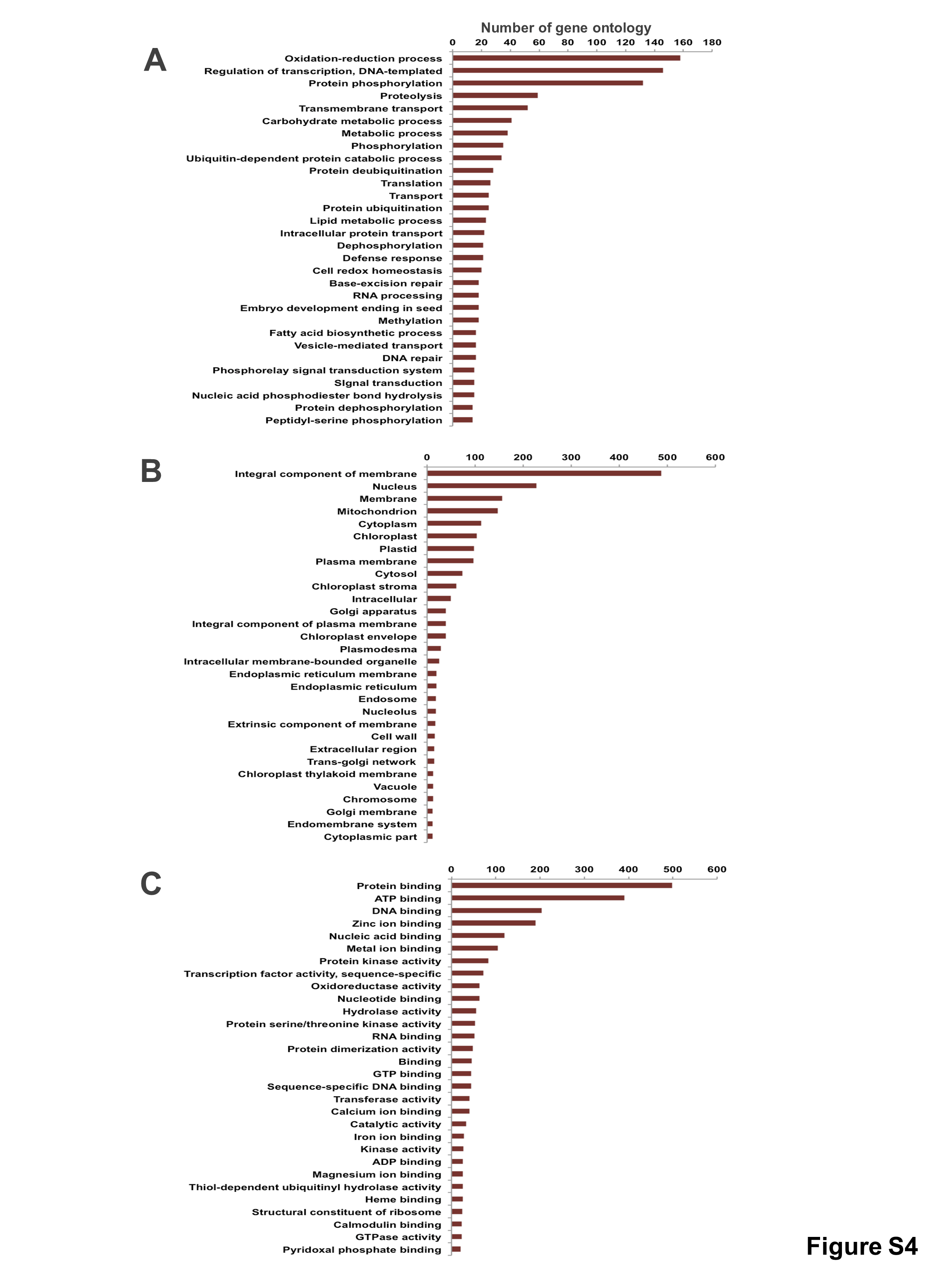

Supplement: S4 Fig — (TIF) [file pone.0191492.s004.TIF]

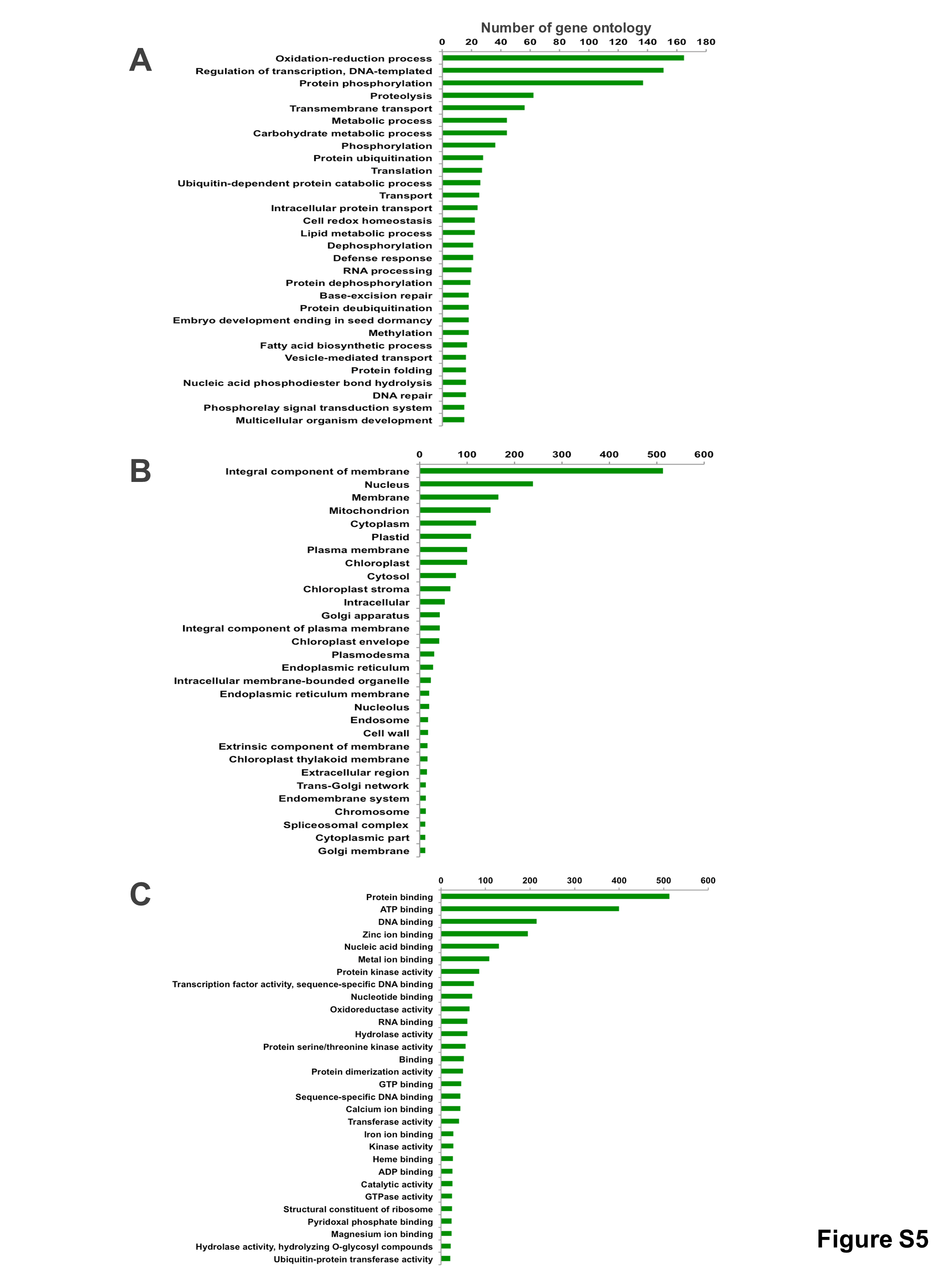

Supplement: S5 Fig — (TIF) [file pone.0191492.s005.TIF]

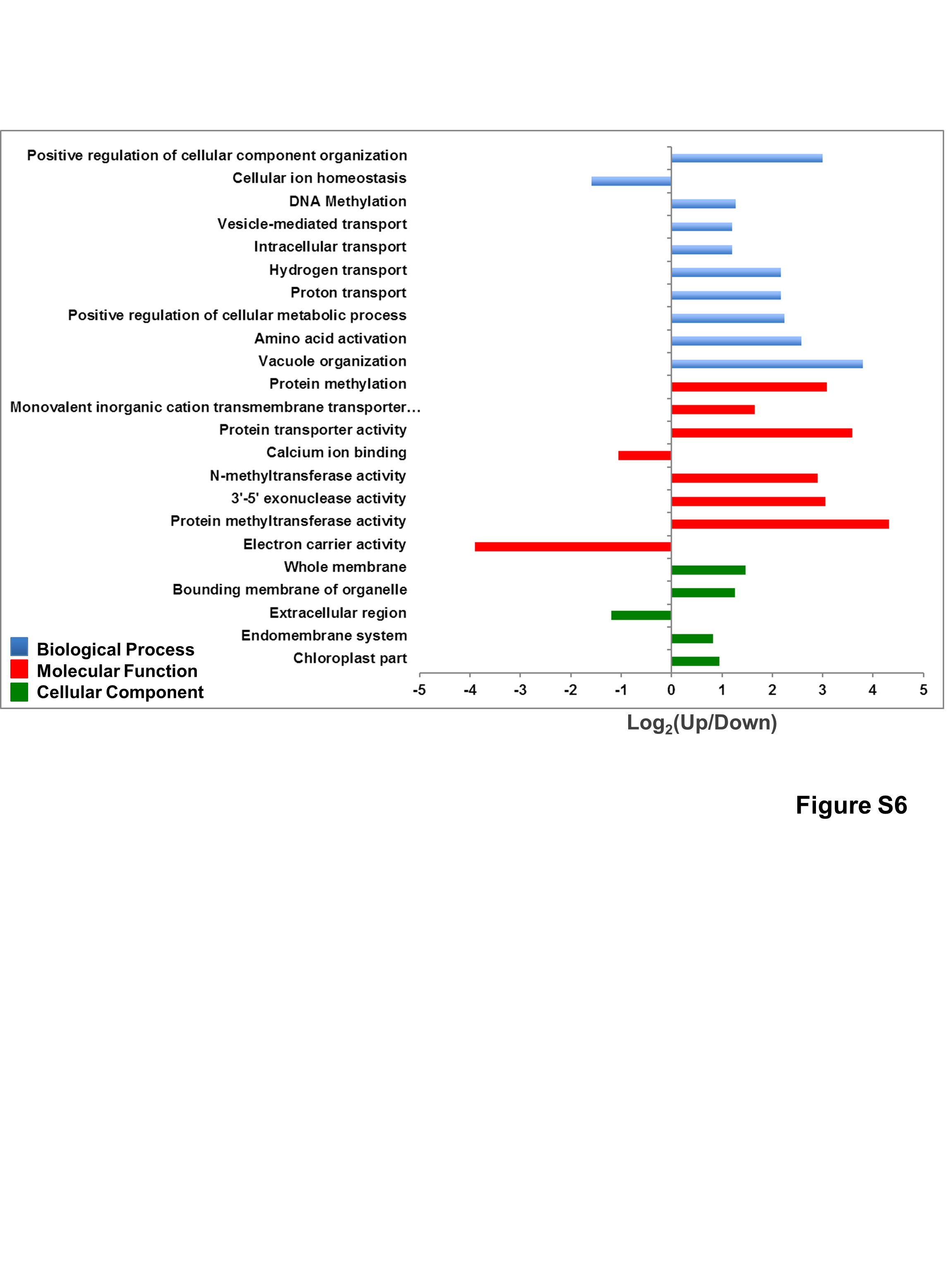

Supplement: S6 Fig — Fisher’s exact test analysis considered the upregulated DMGs as a test group. (TIF) [file pone.0191492.s006.TIF]

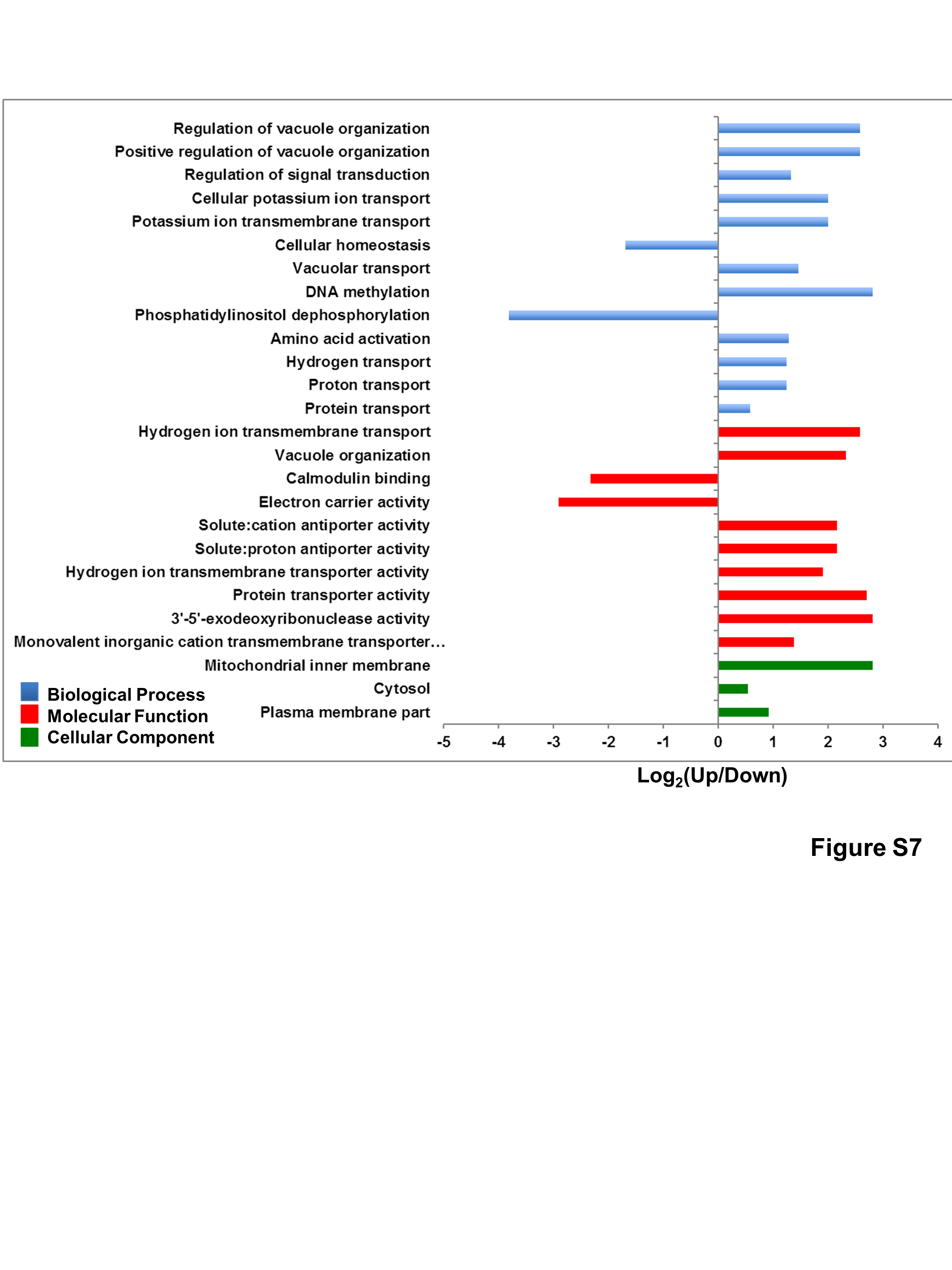

Supplement: S7 Fig — Fisher’s exact test analysis considered the upregulated DMGs as a test group. (TIF) [file pone.0191492.s007.TIF]

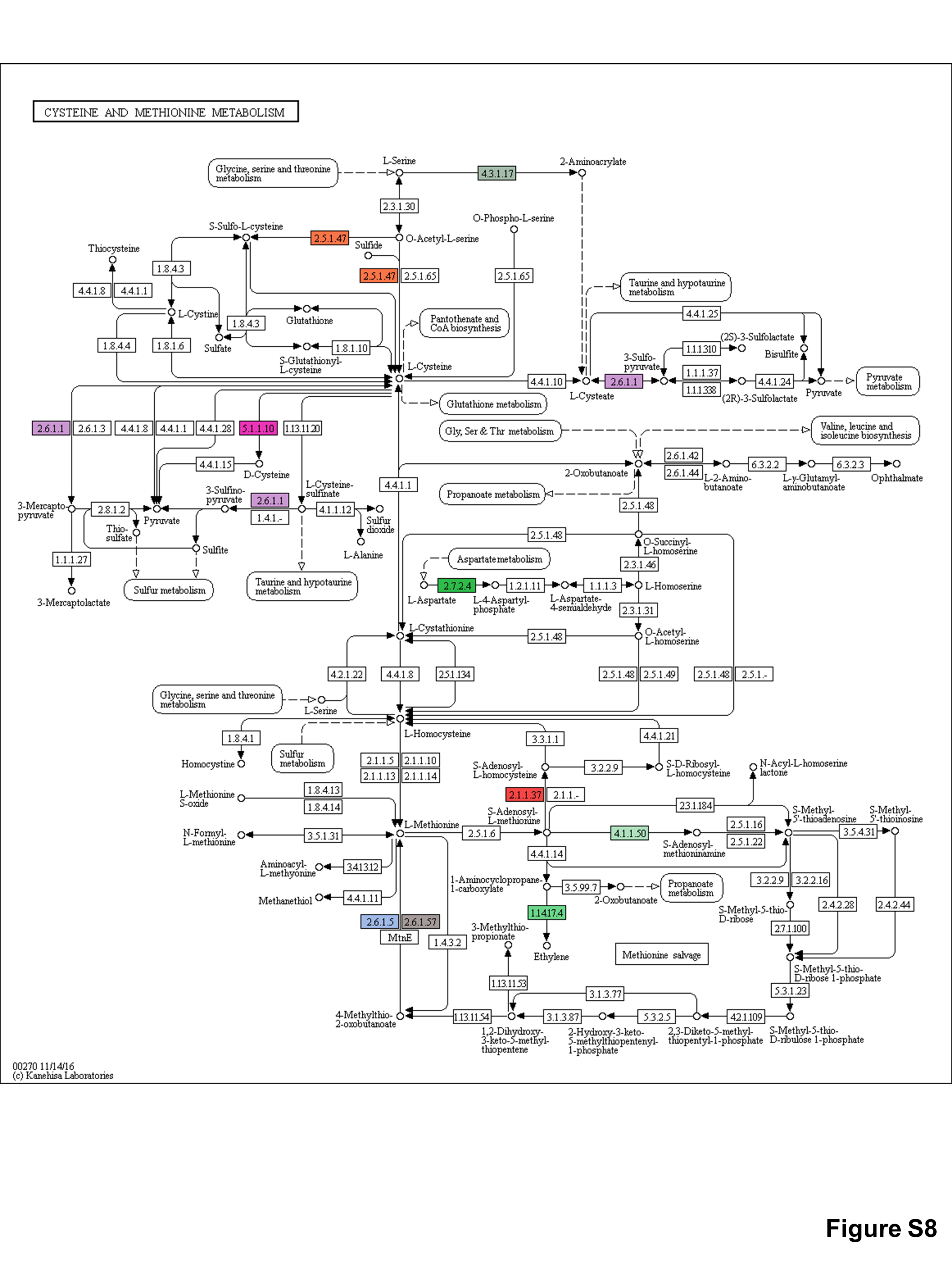

Supplement: S8 Fig — These enzymes might function in the cysteine and methionine metabolic pathway. (TIF) [file pone.0191492.s008.TIF]
